# Hidden Generative AI Use in Consulting: Social-Cognitive and Organizational Factors in AI Disclosure and Concealment

**DOI:** 10.3390/jintelligence14090196

**Published:** 2026-09-01

**Authors:** Filiz Mizrak, Gozde Mert, Ahmet Erkasap, Ali Ozcan, Mehmet Mert

**Affiliations:** 1 Management Information Systems, Atlas Üniversitesi, Istanbul 34408, Turkey; 2 Administrative and Social Sciences, İstanbul Nişantaşı Üniversitesi, Istanbul 34485, Turkey; 3 Business Administration, Istanbul Commerce University, Istanbul 34445, Turkey; 4 Business Administration, İstanbul Nişantaşı Üniversitesi, Istanbul 34485, Turkey

**Keywords:** hidden generative AI use, responsible AI disclosure, consulting, SHAP-XGBoost, Choquet Integral MCDM

## Abstract

Generative artificial intelligence (GenAI) is increasingly embedded in consulting and professional-service work, yet employees may not always disclose its contribution to work outputs. This study examines hidden GenAI use as a social-cognitive, organizational, and human–AI interaction phenomenon and investigates how individual and organizational factors are associated with responsible AI disclosure and concealment. Survey data were collected from 564 consulting and professional-service employees in Türkiye. The analytical framework combined PLS-SEM with covariance-based SEM robustness checks, cross-validated predictive modeling with out-of-fold SHAP interpretation, and exploratory Choquet decision support. The structural results showed that AI disclosure anxiety and competence penalty fear were positively associated with hidden GenAI use, whereas social intelligence, official AI tool adequacy, and responsible AI disclosure were negatively associated with concealment. AI metacognition, human–AI trust, AI policy clarity, social intelligence, and official AI tool adequacy were positively associated with responsible AI disclosure. In the predictive analysis, Ridge regression achieved the strongest average cross-validated performance, while XGBoost was used for nonlinear interpretation through out-of-fold SHAP values; AI disclosure anxiety and competence penalty fear emerged as the strongest predictors. The exploratory Choquet analysis consistently prioritized a non-punitive AI-use disclosure system under alternative weighting and uncertainty scenarios. The study contributes by shifting attention from whether professionals adopt GenAI to whether, why, and under what organizational and social conditions they disclose or conceal its substantive contribution to professional work. The cross-sectional design supports association-based rather than causal interpretation.

## 1. Introduction

Generative artificial intelligence has rapidly become part of everyday knowledge work. In consulting and professional-service organizations, employees increasingly use generative AI tools to draft reports, summarize information, prepare client communications, develop presentations, generate alternatives, and support problem-solving. Unlike earlier workplace technologies that primarily automated routine tasks, generative AI can contribute to writing, reasoning, idea generation, and decision support. This makes it particularly relevant to consulting, where employees are expected to transform complex information into insight, communicate effectively with clients, and produce recommendations under time pressure. The growing use of generative AI reflects a broader shift toward human–AI collaboration. Prior research suggests that AI can support decision-making, creativity, productivity, and task performance when used as an augmentation tool rather than as a substitute for human judgment (Raisch & Krakowski, 2021; Jarrahi et al., 2022; Noy & Zhang, 2023). In consulting, however, the value of AI-supported work still depends on human expertise, contextual judgment, ethical responsibility, and professional accountability. The key issue is therefore not only whether consultants use generative AI but whether they use it critically, transparently, and responsibly.

A central challenge is that AI use may not always be openly disclosed. Consulting work is closely associated with expertise, originality, analytical judgment, and client trust. Employees may therefore worry that revealing AI assistance could make them appear less competent, less original, or overly dependent on technology. When AI contributes to a report, proposal, presentation, or strategic recommendation, disclosure may be interpreted either as responsible transparency or as evidence of reduced professional contribution. This study conceptualizes hidden generative AI use as more than a technological or compliance issue; it may also represent a social and psychological workplace behavior. Employees may conceal AI assistance because they anticipate negative evaluation, competence-related penalties, or damage to their professional image. This logic is consistent with the employee silence literature, which shows that employees may withhold relevant information when expression is perceived as socially or professionally risky (Morrison & Milliken, 2000; Milliken et al., 2003; Van Dyne et al., 2003). In the AI context, the withheld information concerns how a work output was produced, allowing hidden AI use to be examined as a technology-related form of workplace silence. The study also draws on fear of negative evaluation and impression-management perspectives, which suggest that employees actively manage how others perceive their competence and professionalism (Leary, 1983; Bolino & Turnley, 1999). Such pressures may be particularly salient in consulting, where professional value is closely tied to expertise, originality, and independent judgment.

Responsible AI disclosure is also unlikely to depend only on individual willingness. It may be associated with social, emotional, cognitive, and organizational conditions. Social intelligence may help employees judge when and how AI use should be communicated, while emotional intelligence may help regulate disclosure-related anxiety. AI metacognition may support awareness of AI limitations and the significance of its contribution, and human–AI trust may support responsible use when accompanied by verification and professional judgment. Psychological safety, policy clarity, and adequate official AI tools may further reduce uncertainty and make disclosure feel safer and more legitimate. These considerations suggest that hidden AI use should be examined as a human–AI interaction phenomenon embedded within organizational and professional contexts rather than as an isolated individual choice.

Prior research has established several adjacent streams but leaves post-adoption disclosure behavior comparatively underexamined. Studies of generative AI productivity and professional writing emphasize performance benefits (Noy & Zhang, 2023; Brynjolfsson et al., 2025), while research on human–AI augmentation examines how people and intelligent systems complement one another in knowledge work (Raisch & Krakowski, 2021; Jarrahi et al., 2022). Organizational research has focused on adoption, readiness, and governance conditions (Budhwar et al., 2023; Jöhnk et al., 2021), whereas broader responsible-AI scholarship highlights transparency, accountability, and the risks associated with generative AI use (Dwivedi et al., 2023; Wach et al., 2023). These streams explain whether generative AI is adopted, how it affects work, and how organizations may govern its use, but provide less evidence on whether employees disclose meaningful AI assistance once adoption has occurred. The present study addresses this gap by treating hidden generative AI use as a disclosure-related workplace behavior and by distinguishing concealment from AI-use frequency. This distinction is especially relevant in consulting and professional services, where undisclosed AI assistance may raise concerns related to client trust, confidentiality, output reliability, accountability, and professional transparency.

The purpose of this study is to examine and predict hidden generative AI use among consulting and professional-service employees and to explore managerial priorities that may support responsible disclosure. Specifically, the study investigates the associations of social intelligence, emotional intelligence, AI metacognition, psychological safety, human–AI trust, AI policy clarity, official AI tool adequacy, AI disclosure anxiety, and competence penalty fear with responsible AI disclosure and hidden generative AI use. The study makes four substantive contributions. First, it shifts attention from whether professionals adopt generative AI to whether and under what social and organizational conditions they disclose or conceal its meaningful contribution after adoption. Second, it conceptualizes hidden generative AI use as a social-cognitive and human–AI interaction phenomenon linked to workplace silence, evaluation concerns, and professional-image management rather than solely as a compliance problem. Third, it brings individual capabilities and organizational conditions into the same framework, allowing disclosure-related behavior to be examined in relation to social intelligence, emotional intelligence, AI metacognition, human–AI trust, psychological safety, policy clarity, official tool adequacy, disclosure anxiety, and competence-related concerns. Fourth, it provides evidence from consulting and professional services, where AI-supported work is particularly sensitive because professional value is closely tied to expertise, originality, client trust, and accountability.

Methodologically, the study combines explanatory, predictive, and decision-support perspectives. PLS-SEM is used to test the hypothesized relationships, while covariance-based SEM provides an additional robustness assessment of the latent structural model. The predictive stage compares regularized regression, Random Forest, and XGBoost using continuous hidden AI-use scores, with XGBoost further interpreted through out-of-fold SHAP values. Finally, the structural and predictive evidence is incorporated into an exploratory evidence-informed Choquet intervention-prioritization framework. This final stage is intended as decision support rather than as evidence of causal intervention effectiveness. By integrating structural modeling, predictive analysis, explainable machine learning, and exploratory intervention prioritization, the study distinguishes theoretical explanation from predictive importance and managerial prioritization instead of treating these analytical purposes as interchangeable.

The study therefore addresses a timely workplace challenge: how organizations can benefit from generative AI without making its use socially risky to disclose. Responsible AI use requires not only access to technology, but also clear policies, suitable tools, psychologically safe disclosure conditions, and employees capable of evaluating AI outputs critically and communicating their use responsibly. From this perspective, hidden generative AI use is not only a matter of controlling technology; it is also a question of designing organizational conditions that support transparent, accountable, and professionally responsible human–AI collaboration.

## 2. Literature Review and Hypothesis Development

### 2.1. Generative AI Use in Consulting and Professional Services

Generative artificial intelligence has rapidly entered knowledge-intensive work. Unlike earlier forms of workplace automation, generative AI can draft, summarize, compare, translate, classify, recommend, and generate alternative ideas. This makes it highly relevant for consulting and professional services, where employees are expected to prepare reports, presentations, proposals, market analyses, strategic recommendations, and client-facing communication under time pressure. In this context, generative AI is not only a technical tool; it becomes a form of cognitive support that participates in professional reasoning.

Prior research shows that AI is reshaping how organizational tasks are performed and distributed between humans and intelligent systems. Benbya et al. (2020) emphasize that AI creates new organizational opportunities while also requiring new managerial understanding, and Baird and Maruping (2021) explain that employees increasingly delegate tasks to agentic information systems. This is directly relevant to consulting because consultants may delegate early-stage drafting, summarizing, benchmarking, or idea generation to AI while remaining responsible for the final recommendation. However, this delegation changes the meaning of expertise. The consultant is no longer only a producer of knowledge but also an evaluator, editor, verifier, and integrator of AI-generated content.

Consulting is a particularly sensitive context because professional value is closely connected to expertise, analytical judgment, originality, and client trust. Generative AI may help consultants work faster and consider more alternatives, as shown in studies on AI-supported productivity and professional writing (Noy & Zhang, 2023; Brynjolfsson et al., 2025). It may also support creativity and innovation by helping teams generate and refine ideas (Bouschery et al., 2023). Yet these benefits create a practical dilemma. If AI contributes to a client report, proposal, or strategic recommendation, then employees may wonder whether disclosure will reduce the perceived originality or value of their work. Thus, the same technology that improves productivity may also produce anxiety about professional identity and competence.

The human–AI collaboration literature helps explain this tension. Raisch and Krakowski (2021) describe the automation–augmentation paradox, while De Cremer and Kasparov (2021) argue that AI should augment human intelligence rather than replace it. This distinction is central to consulting work. Generative AI can support consultants by structuring information, generating options, or accelerating routine drafting, but it cannot fully replace contextual judgment, client understanding, ethical responsibility, and professional accountability. Similarly, Jarrahi et al. (2022) frame human–AI work as hybrid intelligence, where human and artificial intelligence mutually augment each other. From this perspective, the important question is not simply whether consultants use AI, but whether they use it critically, transparently, and responsibly.

At the same time, generative AI changes the skills expected from professional workers. Bankins et al. (2024) note that AI is reshaping future work skills, and Korzynski et al. (2023) describe prompt engineering as an emerging digital competence. In consulting, this means that expertise increasingly includes the ability to ask effective questions, evaluate AI-generated outputs, recognize errors, and decide when AI assistance is appropriate. Recent published research also emphasizes the importance of organizational readiness, user judgment, and task-specific decisions in effective AI use. Jöhnk et al. (2021) identify strategic alignment, resources, knowledge, culture, and data as central organizational readiness conditions for purposeful AI adoption. Dell’Acqua et al. (2026), in a field experiment involving 758 management consultants, show that generative AI can improve productivity and output quality when tasks fall within its capability frontier but can reduce performance when employees rely on AI for tasks beyond that frontier, highlighting the importance of recognizing AI limitations and exercising professional judgment. Fügener et al. (2026) further demonstrate that effective human–AI collaboration depends on task-specific complementarity and that AI may appropriately automate some tasks, augment humans on others, while certain tasks are better performed by humans without AI support.

However, AI-supported consulting work also introduces organizational and ethical tensions. Algorithms and AI systems may reshape work control, accountability, and collaboration patterns (Faraj et al., 2018; Kellogg et al., 2020; Seeber et al., 2020; Sowa et al., 2021). In consulting firms, employees may feel pressure to produce faster and more polished outputs while also being unsure about whether AI-supported work is acceptable or should be disclosed. Broader discussions of ChatGPT and generative AI highlight similar concerns about reliability, accountability, transparency, and the “dark side” of AI use (Dwivedi et al., 2023; Wach et al., 2023). These concerns are especially important in consulting because AI-supported outputs may influence client decisions.

### 2.2. Hidden Generative AI Use

Hidden generative AI use refers to employees’ intentional concealment of meaningful AI assistance in work-related tasks. In consulting and professional services, this may occur when employees use generative AI to draft reports, summarize data, prepare presentations, generate strategic ideas, refine client communication, or support problem-solving without disclosing that assistance to relevant supervisors, colleagues, or clients. Importantly, hidden AI use should be distinguished from the frequency or intensity of AI use itself. Frequent users may disclose AI assistance consistently, whereas less frequent users may still conceal its contribution when it occurs. The construct therefore concerns transparency regarding meaningful AI-supported work rather than the amount of AI use alone.

The concept can be linked to the broader literature on employee silence. Organizational silence occurs when employees withhold information, concerns, or opinions that could be relevant to organizational functioning (Morrison & Milliken, 2000). Milliken et al. (2003) further show that employees may remain silent when they anticipate negative consequences or perceive speaking up as risky. Although these studies were not developed in the context of generative AI, their underlying logic is applicable to concealed AI use. When employees do not disclose meaningful AI assistance, they withhold information about the process through which a work output was produced rather than about the output itself. In this sense, hidden generative AI use can be understood as a technology-related form of workplace silence.

The distinction between silence and voice proposed by Van Dyne et al. (2003) is also useful in this context. Voice involves the expression of work-related information, whereas silence involves its withholding. Applied to generative AI, disclosure may be interpreted as a form of process transparency because employees communicate the role that AI played in producing a work outcome. Concealment, by contrast, leaves that contribution unspoken. This distinction is particularly relevant in consulting, where deliverables often depend on professional credibility, analytical judgment, originality, and client trust. Concealment should therefore not automatically be interpreted as deliberate deception. Employees may withhold information about AI use because disclosure is perceived as socially risky, professionally ambiguous, or inconsistent with expectations surrounding expertise.

Fear of negative evaluation provides one explanation for such behavior. Leary’s (1983) work suggests that individuals may become concerned about how others will judge them when their competence or performance is made salient. In consulting, these concerns may be intensified because employees are often evaluated on expertise, originality, analytical capability, and problem-solving ability. If consultants believe that acknowledging AI assistance could reduce the perceived value of their own contribution, then concealment may become a means of protecting professional image. This logic is also consistent with impression-management research. Bolino and Turnley (1999) show that employees actively manage how others perceive their competence, commitment, and professionalism. Hidden AI use may therefore function as an impression-management response when AI assistance is perceived as potentially inconsistent with expectations of independent expertise.

Recent AI research reinforces this interpretation. Mirbabaie et al. (2022) show that workplace AI can generate identity-related tensions when employees perceive technology as challenging their professional role or value. Such concerns are particularly relevant in consulting because professional identity is closely tied to expertise, analytical judgment, and the ability to produce credible recommendations. Bankins and Formosa (2023) similarly discuss the implications of AI for meaningful work and human contribution, while Mikalef et al. (2022) emphasize the need to consider the less desirable dimensions of responsible AI adoption. Together, these perspectives suggest that hidden AI use should not be interpreted only as a compliance failure. It may also reflect tensions involving professional identity, autonomy, legitimacy, and the perceived value of human contribution.

The broader risks surrounding generative AI may further complicate disclosure decisions. Wach et al. (2023) highlight concerns related to reliability, accountability, misuse, and ethical uncertainty. These issues are particularly salient in consulting because AI-generated content may be incorporated into client-facing reports, strategic recommendations, proposals, or other decision materials. When employees are uncertain about whether AI use is acceptable, whether its outputs are sufficiently reliable, or whether disclosure is expected, concealment may be associated with ambiguity and uncertainty rather than intentional misconduct alone. This distinction is important because it shifts attention from individual blame toward the organizational conditions under which disclosure decisions are made.

Organizational conditions may therefore be central to understanding hidden AI use. Employees may be more reluctant to disclose AI assistance when policies are unclear, approved tools are inadequate, or informal practices are inconsistent with formal expectations. Research on algorithms and digital technologies at work shows that such systems can reshape power, control, accountability, and employee behavior (Kellogg et al., 2020). Robert et al. (2020) similarly emphasize that fair and responsible AI use depends on appropriate organizational design and governance. In the absence of clear guidance, adequate tools, and psychologically safe conditions, employees may rely on unofficial AI systems or remain uncertain about whether and how their use should be communicated.

Hidden generative AI use is therefore best understood as a social, psychological, and organizational behavior rather than solely as a technological one. It may be associated with fear of negative evaluation, competence-related concerns, unclear organizational rules, insufficient psychological safety, and uncertainty about responsible AI norms. For consulting employees, disclosure decisions are particularly sensitive because they touch directly on professional expertise, judgment, originality, accountability, and trust. Accordingly, the present study examines hidden generative AI use as a form of workplace silence embedded within individual, social, cognitive, and organizational conditions.

### 2.3. Social and Emotional Intelligence in Human–AI Interaction

The decision to disclose or hide generative AI use is not only a technical decision. It is also a social and emotional decision. In consulting and professional services, employees must judge how supervisors, colleagues, and clients may interpret their work process. If AI assistance is disclosed, then others may see the employee as transparent and responsible. However, they may also question the employee’s originality, expertise, or analytical contribution. Therefore, AI disclosure takes place within a social context where employees must interpret expectations, manage impressions, and regulate their emotional responses.

Social intelligence is particularly relevant in this context because it reflects the ability to understand social situations, interpret others’ expectations, and respond appropriately. Silvera et al. (2001) conceptualize social intelligence as an ability related to social information processing, social skills, and social awareness. In consulting work, these abilities may help employees understand when AI use should be disclosed, how disclosure should be communicated, and how different audiences may respond. For example, disclosing AI assistance to a project team may require a different communication style than disclosing it to a client. A socially intelligent consultant may be better able to frame AI use as responsible support rather than as a weakness in professional capability. Therefore, this study proposes that social intelligence negatively affects hidden generative AI use (H1) and positively affects responsible AI disclosure (H2).

Emotional intelligence is also central to AI disclosure behavior. Wong and Law (2017) describe emotional intelligence as the ability to understand and regulate one’s own emotions and to understand the emotions of others. In AI-supported consulting work, employees may experience anxiety, embarrassment, or uncertainty when deciding whether to disclose AI assistance. This anxiety may be stronger when employees believe that AI use could make them appear less competent or less original. Emotional intelligence may help employees manage these concerns, remain calm during evaluation, and make more balanced disclosure decisions. Accordingly, this study proposes that emotional intelligence negatively affects AI disclosure anxiety (H3).

The role of emotional intelligence is especially important because AI disclosure anxiety is closely related to fear of negative evaluation. Leary (1983) explains that individuals may become anxious when they expect others to evaluate them unfavorably. In the present context, employees may worry that supervisors, colleagues, or clients will judge them negatively if they reveal that generative AI contributed to their work. Bolino and Turnley’s (1999) work on impression management also supports this argument, as employees often attempt to protect a desired professional image. In consulting, where image, expertise, and client trust are highly visible, employees may hide AI use to avoid appearing dependent on technology. Therefore, this study proposes that AI disclosure anxiety positively affects hidden generative AI use (H4).

Prior studies on human responses to intelligent technologies further suggest that social and emotional perceptions shape how people interact with AI. Dang and Liu (2022) show that people’s beliefs about the human mind can influence cooperative or competitive responses to AI robots, while Yam et al. (2021) indicate that individuals respond differently to intelligent agents depending on perceived social and emotional qualities. Although these studies focus on AI and robots more broadly, they support the idea that AI use is not evaluated in purely rational terms. People respond to AI through social meanings, emotional reactions, and judgments about human capability.

In consulting work, generative AI disclosure is therefore not simply a question of following a rule. It is a socially sensitive act. Employees must decide whether disclosure will be seen as honesty, weakness, efficiency, or lack of originality. Social intelligence may help them navigate this interpersonal complexity, while emotional intelligence may help them regulate the anxiety attached to possible negative evaluation. These social and emotional mechanisms provide an important foundation for understanding why some consulting employees disclose AI assistance responsibly, whereas others conceal it.

### 2.4. AI Metacognition, Human–AI Trust, and Responsible AI Disclosure

Responsible use of generative AI in consulting requires more than technical access to AI tools. Consultants must also understand what AI can and cannot do, when AI-generated content should be checked, and when human professional judgment should override machine-generated suggestions. In this study, AI metacognition refers to employees’ awareness of the strengths, limitations, and risks of generative AI outputs. It reflects the ability to think about one’s own AI-supported thinking process. A consultant with stronger AI metacognition does not simply accept AI output as ready-to-use content; instead, they evaluate whether the output is accurate, relevant, ethical, and appropriate for the client or project context.

The concept of AI metacognition is grounded in broader metacognitive awareness research. Schraw and Dennison (1994) describe metacognition as awareness and regulation of one’s own cognitive processes. In the context of generative AI, this awareness becomes especially important because AI can produce fluent and persuasive outputs even when the content is incomplete, biased, or inaccurate. AI literacy research similarly emphasizes that users need to understand, evaluate, and critically engage with AI systems rather than use them passively (Long & Magerko, 2020; Ng et al., 2021; Wang et al., 2023). For consulting employees, this means knowing when AI is useful for drafting, summarizing, or generating alternatives, but also knowing when AI output requires verification, contextual interpretation, or rejection.

This metacognitive capacity is closely related to responsible AI disclosure. Employees who understand the limitations and risks of generative AI are more likely to recognize when AI assistance is meaningful enough to be disclosed. For example, if AI only helped improve grammar, then disclosure may be less critical; if it shaped a strategic recommendation, then disclosure may become more important. AI metacognition therefore helps employees distinguish between minor technical support and substantive AI contribution. In consulting work, where client trust and professional accountability matter, this awareness can support more transparent and responsible AI behavior. Accordingly, this study proposes that AI metacognition positively affects responsible AI disclosure (H5).

AI metacognition may also shape human–AI trust. Human–AI trust refers to employees’ confidence in AI tools and their willingness to rely on them for work-related support. Earlier trust-in-automation research shows that trust is central to how people interact with automated systems (Jian et al., 2000), and more recent work confirms that trust plays an important role in AI acceptance and use (Choung et al., 2023; Glikson & Woolley, 2020). However, trust in AI should not be understood as blind confidence. In consulting work, excessive trust may lead employees to accept AI-generated content without sufficient review, while very low trust may prevent them from using AI even when it could provide useful support.

For this reason, balanced trust is important. Employees with stronger AI metacognition may develop a more calibrated form of trust because they understand both the value and limitations of generative AI. They may trust AI for tasks such as drafting, summarizing, or idea generation, but remain cautious when the output involves factual accuracy, ethical judgment, client-specific interpretation, or strategic recommendations. This balanced trust is closer to responsible professional use than either overreliance or rejection. Therefore, this study proposes that AI metacognition positively affects human–AI trust (H6).

Human–AI trust may also support responsible AI disclosure. When employees trust AI as a legitimate support tool, they may feel less need to hide its use. In contrast, if AI use is seen as unreliable, inappropriate, or professionally questionable, then employees may become more hesitant to disclose it. Trust can therefore make AI use feel more acceptable and discussable, especially when employees believe that AI can add value without replacing their professional judgment. Studies on human–AI collaboration suggest that effective AI use depends on the ability to coordinate human expertise and machine support rather than treating AI as either a threat or a substitute for human capability (Fügener et al., 2021; Jarrahi et al., 2022; Raisch & Krakowski, 2021).

Explainable AI research further supports this logic. Barredo Arrieta et al. (2020), Rai (2020), and Meske et al. (2022) argue that transparency and explainability are important for responsible AI use. In the present study, disclosure functions as a human-centered form of transparency. It allows employees to communicate when AI contributed to a work output and to remain accountable for how that output was developed. In consulting, this is particularly important because AI-supported work may influence client decisions. Thus, when human–AI trust is balanced and supported by metacognitive awareness, employees are more likely to disclose AI assistance responsibly rather than conceal it. Accordingly, this study proposes that human–AI trust positively affects responsible AI disclosure (H7).

AI metacognition and human–AI trust are central to understanding responsible generative AI use in consulting. Consultants need to know not only how to use AI tools, but also how to evaluate their outputs, recognize their risks, and communicate their role in professional work. Responsible AI disclosure is therefore not merely a compliance behavior; it is a form of reflective professional judgment in human–AI interaction.

### 2.5. Psychological Safety, AI Policy Clarity, and Official AI Tool Adequacy

Hidden generative AI use is not shaped only by individual attitudes or skills. It is also influenced by the organizational environment in which employees decide whether AI use is safe to disclose. In consulting and professional services, this environment is especially important because employees often work under client pressure, performance expectations, and strong norms of professional expertise. Even if employees understand how to use generative AI responsibly, they may still hide AI assistance if the workplace climate makes disclosure feel risky.

Psychological safety is central to this issue. Edmondson (1999) defines psychological safety as a shared belief that employees can ask questions, admit uncertainty, and discuss work-related concerns without fear of punishment or embarrassment. In the context of consulting work, psychological safety may determine whether employees feel comfortable explaining how they completed a task, including whether generative AI supported part of the process. When psychological safety is high, employees are more likely to discuss uncertainty, ask for guidance, and disclose technology-supported work. When it is low, employees may fear that AI use will damage their professional image or make them appear less capable.

This fear is closely related to competence penalty fear. Consulting employees are often evaluated based on analytical ability, judgment, originality, and client-facing professionalism. If they believe that AI use will make others question these qualities, then they may avoid disclosure even when AI use was reasonable or helpful. O’Donovan and McAuliffe (2020) show that psychological safety is enabled by supportive leadership, interpersonal trust, and open communication, all of which are relevant for reducing fear in professional teams. Therefore, this study proposes that psychological safety negatively affects competence penalty fear (H8). In turn, when employees expect a competence penalty from AI disclosure, they may conceal AI assistance to protect their professional image. Accordingly, this study proposes that competence penalty fear positively affects hidden generative AI use (H9).

Beyond team climate, formal organizational guidance also matters. AI policy clarity refers to the extent to which an organization provides understandable rules about when, how, and for what purposes generative AI can be used. In consulting, policy clarity is particularly important because AI-supported outputs may be included in client reports, proposals, presentations, or strategic recommendations. If employees do not know whether AI use is allowed, whether it must be disclosed, or what types of information can be entered into AI tools, then they may become anxious about revealing AI assistance. Budhwar et al. (2023) emphasize that generative AI requires new HR and managerial frameworks, while Mikalef et al. (2022) argue that responsible AI requires attention to the darker and riskier sides of AI use. These studies suggest that organizations cannot simply encourage AI adoption without clarifying responsible-use boundaries.

Clear policies may reduce anxiety because they make acceptable behavior easier to understand. If employees know when AI use is permitted and how disclosure should occur, then AI use becomes less socially and professionally ambiguous. In contrast, unclear rules may lead employees to rely on personal judgment, informal norms, or hidden practices. Research on organizational readiness for AI also shows that AI adoption depends on structural and managerial conditions, not only individual willingness (Jöhnk et al., 2021). Accordingly, this study proposes that AI policy clarity negatively affects AI disclosure anxiety (H10) and positively affects responsible AI disclosure (H11).

Official AI tool adequacy is another important organizational condition. It refers to whether employees have access to approved, secure, and task-relevant AI tools that can support their work. If consulting employees do not have adequate official tools, then they may use external or unofficial AI systems to complete tasks more efficiently. However, unofficial use may be harder to disclose because it may involve uncertainty about data security, client confidentiality, or compliance with organizational expectations. In this way, inadequate official tools may unintentionally encourage shadow AI use and concealment.

Prior research on AI in organizations suggests that responsible adoption requires more than enthusiasm for innovation. Robert et al. (2020) argue that fair and responsible AI use depends on careful organizational design, while Parker and Grote (2022) emphasize that digital work design matters in algorithmic and automated environments. These arguments suggest that employees need not only permission to use AI, but also reliable tools and supportive structures that make responsible use practical. Therefore, this study proposes that official AI tool adequacy negatively affects hidden generative AI use (H12) and positively affects responsible AI disclosure (H13).

Taken together, psychological safety, AI policy clarity, and official AI tool adequacy represent the organizational side of responsible generative AI use. Employees are less likely to disclose AI assistance when they fear competence loss, when rules are unclear, or when they must rely on unofficial tools. Conversely, a psychologically safe environment, clear AI-use policies, and adequate official tools can make AI disclosure more acceptable, less risky, and more professionally responsible.

### 2.6. Responsible AI Disclosure and Hidden Generative AI Use

Responsible AI disclosure refers to employees’ willingness to communicate meaningful AI assistance when such transparency is appropriate. In consulting and professional services, this does not imply that every minor grammar correction, formatting suggestion, or low-level technical use of AI must be disclosed. Rather, disclosure becomes particularly relevant when generative AI makes a substantive contribution to a report, proposal, presentation, client communication, analytical process, or strategic recommendation. In such cases, employees should be able to explain the role of AI clearly and responsibly. This is especially important in consulting, where clients and managers evaluate not only the final deliverable but also the professional judgment, reasoning, and accountability underlying it. When AI contributes meaningfully to a work output but its role remains invisible, uncertainty may arise regarding how the recommendation was developed, who exercised judgment, and whether the output was appropriately reviewed.

The literature on employee voice and silence provides a useful framework for understanding this behavior. Voice refers to the expression of relevant work-related information, ideas, or concerns, whereas silence refers to withholding such information (Van Dyne & LePine, 1998; Van Dyne et al., 2003). From this perspective, responsible AI disclosure can be understood as a form of process transparency or voice in AI-supported work. When employees disclose meaningful AI assistance, they make an important aspect of the production process visible. When they conceal that assistance, they remain silent about how the work was produced. However, concealment should not automatically be interpreted as deliberate deception. Employees may be uncertain about whether disclosure is expected, how it will be received, or whether acknowledging AI support could damage their professional image.

Responsible AI disclosure and hidden generative AI use are therefore treated in this study as related but distinct behaviors rather than as exact opposites on a single continuum. Responsible disclosure reflects an affirmative willingness to communicate meaningful AI assistance when transparency is appropriate, whereas hidden AI use reflects the withholding of such information. Employees may exhibit different levels of disclosure and concealment across tasks, audiences, or organizational situations. For example, an employee may disclose AI assistance in an internal analytical task but remain reluctant to disclose similar assistance in a client-facing deliverable. This distinction allows transparency behavior and concealment behavior to be examined separately while recognizing that they are expected to be negatively related.

The broader literature on responsible AI further emphasizes transparency, accountability, and explainability as central elements of trustworthy AI use (Barredo Arrieta et al., 2020; Mikalef et al., 2022; Rai, 2020). In the present study, disclosure is treated as a human-centered form of transparency. It involves more than simply stating that “AI was used”; it concerns clarifying how AI contributed, how its output was evaluated, and where human professional judgment remained central. This is particularly important in consulting because AI-supported outputs may influence client decisions, strategic recommendations, and organizational actions. Explainable AI research similarly emphasizes the importance of understanding how AI contributes to outputs and decisions (Lundberg et al., 2020; Meske et al., 2022; Samek et al., 2021). Although this literature often focuses on technical explainability, the underlying principle is also relevant to everyday professional practice, where employees may need to explain how AI-generated content was checked, evaluated, and integrated into a final deliverable.

Responsible disclosure may also support organizational learning. Morrison and Milliken (2000) argue that silence can prevent organizations from accessing information that is important for adaptation and improvement. A similar logic applies to generative AI use. When employees openly communicate how they are using AI, organizations are better positioned to identify beneficial applications, recognize emerging risks, develop appropriate guidance, and improve governance practices. When AI use remains hidden, organizations may have less visibility into where AI is supporting work, where it may create accountability or quality concerns, and what kinds of policies or training employees require.

Accordingly, the present study proposes that responsible AI disclosure is negatively associated with hidden generative AI use. Employees who are more willing to communicate meaningful AI assistance are expected to report lower levels of concealment. This relationship is stated as a theoretically directional hypothesis, while recognizing that the cross-sectional design does not establish temporal or causal ordering. Accordingly, the present study proposes that responsible AI disclosure is negatively associated with hidden generative AI use (H14).

Responsible AI disclosure is conceptualized not as a simple compliance requirement but as a professional practice linked to transparency, accountability, and responsible human–AI interaction. In consulting and professional services, such disclosure may help make AI-supported work more visible and governable while preserving the central role of human judgment and professional responsibility.

## 3. Methodology

### 3.1. Research Design and Sample

This study employed a quantitative, cross-sectional survey design to examine hidden generative AI use among employees working in consulting and professional services in Türkiye. Data were collected between 20 March and 20 April 2026 through an anonymous online questionnaire administered via Google Forms. Participants were recruited using purposive and professional-network sampling through LinkedIn, professional contacts, and company networks. Eligibility required respondents to be 18 years of age or older, currently employed in Türkiye in consulting or professional-service work, and to have prior experience using generative AI for work-related tasks. The targeted recruitment strategy was intended to ensure that participants had relevant professional exposure to AI-supported knowledge work. Approximately 920 professionals were approached, yielding a final analytical sample of 564 usable responses. Because the number of individuals who opened or started the survey was not retained, the resulting 61.3% figure is best interpreted as an approximate usable-response yield rather than a conventional response rate. Participation was voluntary, no financial or material incentives were offered, and no names, email addresses, or company-identifying information were collected. No formal a priori power analysis was used; the sampling objective was to obtain a sufficiently large professional sample for stable estimation of the latent-variable and predictive models. The final sample of 564 provided sufficient observations for the planned multivariate analyses, repeated cross-validation, and subgroup sensitivity checks. Because survey-start counts, incomplete-response dispositions, and characteristics of nonrespondents were not retained, a formal nonresponse-bias analysis and an exact exclusion count could not be reconstructed; this limitation is considered when interpreting the representativeness of the sample.

The final sample included respondents working in management consulting, strategy consulting, business analysis, IT consulting, HR consulting, financial consulting, audit and advisory, market research, and related professional-service areas. The consulting context was selected because professional-service work relies heavily on analytical judgment, expertise, originality, client communication, and trust, making it particularly suitable for examining transparency and concealment surrounding AI-supported work. All respondents had prior work-related experience with generative AI tools such as ChatGPT, Gemini, Copilot, Claude, or similar systems. The questionnaire measured social intelligence, emotional intelligence, AI metacognition, psychological safety, human–AI trust, AI policy clarity, official AI tool adequacy, AI disclosure anxiety, competence penalty fear, responsible AI disclosure, and hidden generative AI use.

The analytical workflow comprised four complementary stages. First, the measurement model was evaluated using reliability, convergent- and discriminant-validity diagnostics, covariance-based confirmatory factor analysis, and common-method diagnostics. Second, the hypothesized structural model was estimated using PLS-SEM and covariance-based SEM as a robustness check. Third, hidden generative AI use was modeled primarily as a continuous outcome using cross-validated predictive models, with XGBoost interpreted through out-of-fold SHAP values; binary HGAI classifications were retained only as threshold-sensitivity analyses. Finally, an exploratory evidence-informed Choquet intervention-prioritization framework was used to compare managerial alternatives under alternative weighting and uncertainty scenarios. The structural, predictive, and decision-support stages were interpreted as complementary rather than causally equivalent.

### 3.2. Measures

The questionnaire comprised 57 Likert-type items measuring eleven constructs. All items were assessed on a five-point scale ranging from 1 = strongly disagree to 5 = strongly agree. Established scales were adapted to the context of generative AI use in consulting and professional services where appropriate, while context-specific items were used for AI policy clarity and official AI tool adequacy. Before the main data collection, the questionnaire was reviewed by four academics to assess conceptual relevance, wording clarity, and contextual appropriateness. A pilot study involving 30 participants was subsequently conducted to evaluate item comprehensibility, questionnaire flow, terminology, and overall usability. Based on this process, minor wording and presentation refinements were made without altering the substantive meaning or dimensional structure of the measures. The questionnaire was administered in English; therefore, no translation or back-translation procedure was required.The measurement structure is summarized in Table 1. The complete item wording and source basis are provided in Supplementary Table S1.

Social intelligence was assessed using five items adapted from Silvera et al. (2001), while emotional intelligence was measured with five items adapted from Wong and Law (2017). AI metacognition was assessed using six items informed by Schraw and Dennison (1994) and Ng et al. (2021), focusing on respondents’ awareness of the strengths, limitations, and risks of AI-supported reasoning. Psychological safety was measured using five items adapted from Edmondson (1999), and human–AI trust was assessed using five items based on Jian et al. (2000) and Choung et al. (2023).

AI policy clarity and official AI tool adequacy were each measured with five context-adapted items. AI policy clarity captured the extent to which organizational rules concerning acceptable AI use and disclosure were perceived as understandable and explicit, whereas official AI tool adequacy reflected the perceived availability, security, and task suitability of organization-approved AI tools. Particular attention was given during academic review and pilot testing to the clarity and relevance of the wording of these items.

AI disclosure anxiety was assessed using five items adapted from Leary (1983), while competence penalty fear was measured using five items informed by Leary (1983) and Bolino and Turnley (1999). Responsible AI disclosure was assessed with five items adapted from the employee voice literature (Van Dyne & LePine, 1998; Van Dyne et al., 2003), and hidden generative AI use was measured using six items informed by organizational silence research (Morrison & Milliken, 2000; Milliken et al., 2003; Van Dyne et al., 2003). Responsible AI disclosure and hidden generative AI use were modeled as related but distinct constructs: the former reflects affirmative transparency regarding meaningful AI assistance, whereas the latter reflects the withholding of such information.

Before testing the structural model, the measurement properties of the scales were assessed. The Kaiser–Meyer–Olkin measure was 0.909, indicating excellent sampling adequacy. Bartlett’s test of sphericity was significant, χ^2^(1596) = 12,997.215, *p* < .001, confirming that the correlation matrix was suitable for factor-based analysis. Construct-level distribution and missingness diagnostics and item-level measurement results are provided in Supplementary Tables S2 and S3.

Table 2 summarizes the measurement properties. Internal consistency was adequate across all eleven constructs, with Cronbach’s alpha values ranging from 0.798 to 0.858 and omega/composite reliability values ranging from 0.799 to 0.858. Standardized CFA loadings ranged from 0.599 to 0.807. The eleven-factor CFA demonstrated excellent global fit. Several CFA-based AVE values were marginally below the conventional 0.50 guideline; however, these constructs were retained because their reliability coefficients and standardized loadings remained acceptable and the broader discriminant-validity evidence supported their empirical distinctiveness. Accordingly, convergent validity was interpreted cautiously rather than treated as uniformly strong across all constructs.

Discriminant validity was assessed using HTMT, latent correlations, and construct-specific CFA comparisons. All HTMT values remained below the conservative 0.85 criterion. In particular, responsible AI disclosure and hidden generative AI use demonstrated clear empirical distinctiveness, supporting their treatment as related but separate constructs rather than opposite ends of a single continuum.Bootstrap HTMT confidence intervals and the Fornell–Larcker matrix are reported in Supplementary Table S4. HTMT interpretation followed the criterion proposed by Henseler et al. (2015).

## 4. Analytical Procedure

The analytical procedure integrates four linked components: measurement-model assessment; structural modeling using PLS-SEM with CB-SEM robustness checks; prediction of continuous hidden generative AI use using benchmark models, cross-validation, and out-of-fold SHAP interpretation; and exploratory evidence-informed Choquet intervention prioritization. The intervention stage combines empirically derived criterion importance with an explicit scenario-scoring rubric and is interpreted as a decision-support exercise rather than as causal evidence of intervention effectiveness.

**Figure 1:**
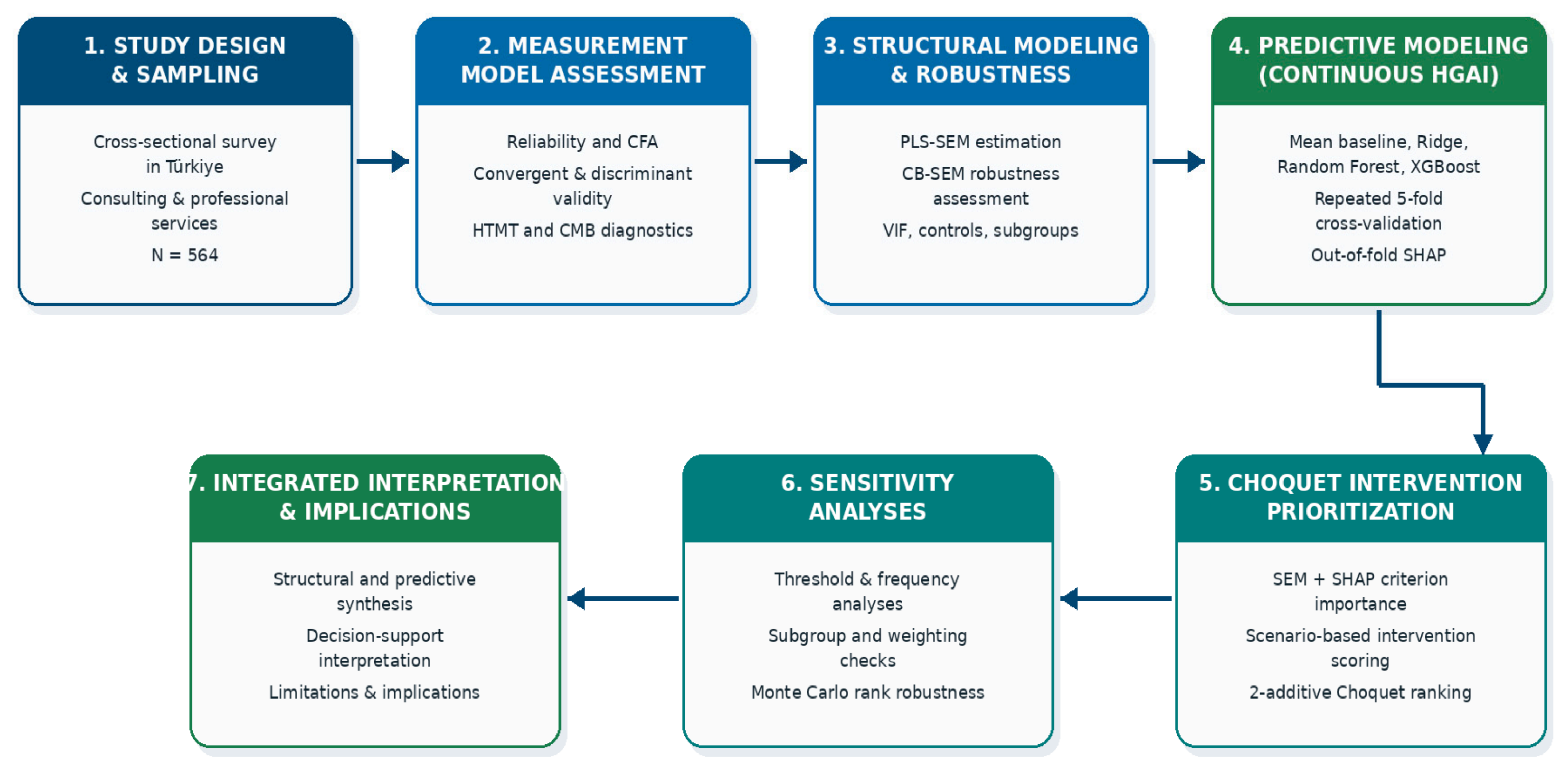
Analytical workflow of the study.

The overall analytical workflow is presented in Figure 1. The framework distinguishes explanatory structural modeling from predictive modeling and treats the Choquet stage as exploratory decision support. Sensitivity analyses are incorporated to assess the stability of the structural, predictive, and intervention-prioritization results before the findings are integrated and interpreted.

### 4.1. Structural Modeling and Robustness Assessment

Partial least squares structural equation modeling (PLS-SEM) was retained as the primary structural estimator because the study combines explanatory structural modeling with a subsequent prediction-oriented analytical stage and uses construct-level structural information in the intervention-prioritization framework. Given the adequate sample size and the reflective nature of the measures, covariance-based SEM was additionally estimated for the same theoretical model to evaluate global fit and determine whether the substantive structural conclusions depended on the PLS estimator. The PLS-SEM analysis was conducted in SmartPLS. The measurement and structural components were evaluated separately so that the adequacy of the latent constructs could be established before interpreting the hypothesized structural relationships. This use of PLS-SEM within an explanatory–predictive workflow is consistent with methodological guidance on PLS-SEM reporting and application (Hair et al., 2019; Sarstedt et al., 2022).

For completeness, the measurement and structural diagnostics used in the PLS-SEM assessment were calculated using the following expressions.

(1) $$AVE=\frac{\sum_{i=1}^{k}\lambda_{i}^{2}}{k}$$

where *λ_i_* is the standardized loading of item *i* and *k* is the number of indicators.

(2) $$CR=\frac{{\left(\sum_{i=1}^{k}\lambda_{i}\right)}^{2}}{{\left(\sum_{i=1}^{k}\lambda_{i}\right)}^{2}+\sum_{i=1}^{k}\left(1-\lambda_{i}^{2}\right)}$$

where *λ_i_* denotes the standardized indicator loading and 1 − λ_i_^2^ represents indicator error variance.

(3) $${HTMT}_{ab}=\frac{\frac{1}{m_{a}m_{b}}\sum_{i=1}^{m_{a}}\sum_{j=1}^{m_{b}}\left|r_{ij}\right|}{\sqrt{\left(\frac{1}{m_{a}\left(m_{a}-1\right)}\sum_{i\neq j}^{m_{a}}\left|r_{ij}\right|\right)\left(\frac{1}{m_{b}\left(m_{b}-1\right)}\sum_{i\neq j}^{m_{b}}\left|r_{ij}\right|\right)}}$$

where r*_ij_* denotes the correlation between indicators and *m_a_* and *m_b_* denote the number of indicators in constructs *a* and *b*, respectively.

The structural model was assessed using standardized path coefficients, bootstrap-based significance tests, coefficients of determination (*R*^2^), effect-size estimates, predictive relevance, and collinearity diagnostics. Structural variance inflation factors (VIFs) were examined to determine whether the estimated paths were affected by problematic overlap among predictors. The direction, magnitude, and statistical uncertainty of the hypothesized paths were interpreted jointly rather than relying on statistical significance alone.

(4) $$\eta_{j}=\sum_{n=1}^{N}\beta_{jn}\xi_{n}+\zeta_{j}$$

where *η_j_* is an endogenous construct, *ξ_n_* represents predictor constructs, *β_jn_* denotes the structural coefficient, and *ζ*_j_ is the residual term.

(5) $$R^{2}=1-\frac{SS_{res}}{SS_{tot}}$$

where *SS*_res_ is the residual sum of squares and *SS*_tot_ is the total sum of squares.

(6) $$f^{2}=\frac{R_{included}^{2}-R_{excluded}^{2}}{1-R_{included}^{2}}$$

The *f*^2^ statistic expresses the contribution of a predictor by comparing *R*^2^ with and without that predictor.

(7) $$Q^{2}=1-\frac{PRESS}{SSO}$$

where PRESS is the prediction error sum of squares and SSO is the sum of squares of observations.

Covariance-based structural equation modeling (CB-SEM) was used as an independent robustness assessment of the latent structural model. The same eleven-construct measurement structure and hypothesized structural relationships were estimated using covariance-based SEM, and global fit was evaluated using the chi-square statistic, comparative fit index (CFI), Tucker–Lewis index (TLI), root mean square error of approximation (RMSEA), and standardized root mean square residual (SRMR). Consistency in coefficient direction and substantive interpretation across PLS-SEM and CB-SEM was used as evidence that the conclusions were not dependent on a single SEM estimator.

Additional structural checks addressed alternative explanations raised by the theoretical model. The relationship between AI metacognition and responsible AI disclosure was examined both directly and through human–AI trust, and an exploratory AI metacognition × human–AI trust interaction was tested for responsible AI disclosure. Models including AI familiarity, AI-use frequency, formal AI policy, responsible-AI training, and client-communication involvement were estimated to assess whether the focal structural relationships remained stable after accounting for relevant background conditions.

Generalizability across work contexts was examined through subgroup analyses. Respondents who reported using generative AI for client communication were compared with those who did not, and an additional professional-area comparison examined support-oriented IT/HR roles (*n* = 152) and client/advisory-oriented roles (*n* = 389); 23 respondents classified as “Other” were excluded from this particular comparison because they could not be assigned unambiguously to either subgroup. Path differences were interpreted as exploratory because subgroup classification was based on observed work characteristics rather than randomized assignment. Plausible reverse-direction specifications were also estimated as sensitivity checks. These models were not treated as competing causal demonstrations; instead, they were used to assess the extent to which the cross-sectional data could support alternative directional associations and to guide appropriately cautious interpretation.

### 4.2. Predictive Modeling and Out-of-Fold SHAP Interpretation

The predictive stage examined hidden generative AI use as a continuous outcome. The primary outcome was the arithmetic mean of the retained HGAI items, preserving the full variation of the construct rather than imposing a single binary risk threshold. Predictor values were calculated as arithmetic mean composite scores for social intelligence, emotional intelligence, AI metacognition, psychological safety, human–AI trust, AI policy clarity, official AI tool adequacy, AI disclosure anxiety, and competence penalty fear. Responsible AI disclosure was excluded from the primary predictor set because of its conceptual proximity to concealment and was introduced only in a separate sensitivity specification.

Predictive performance was evaluated by comparing a mean baseline, Ridge regression, random forest, and XGBoost. Repeated five-fold cross-validation was used for the broad model comparison, and out-of-fold predictions were retained so that performance estimates were based on observations not used to fit the corresponding model. Mean absolute error (MAE), root mean square error (RMSE), and R^2^ were used as the principal continuous-outcome performance metrics. XGBoost hyperparameters were selected within the training folds from a prespecified search space to limit optimistic performance estimates. Fixed random seeds were used consistently for cross-validation partitioning and stochastic model estimation to support reproducibility.

For XGBoost, the continuous HGAI prediction function was represented as the additive contribution of T regression trees:

(8) $${\hat{y}}_{i}=\sum_{t=1}^{T}f_{t}\left(x_{i}\right),\;f_{t}\in F$$

where *ŷ_i_* is the predicted HGAI value for respondent *i*, *x_i_* is the predictor vector, and *f_t_* denotes tree *t*.

(9) $$Obj=\sum_{i=1}^{n}l\left(y_{i},{\hat{y}}_{i}\right)+\sum_{t=1}^{T}\Omega \left(f_{t}\right)$$

where l(·) is the loss function and Ω(*f_t_*) is the regularization term controlling model complexity.

XGBoost was retained as the nonlinear explainable model even when a simpler benchmark achieved stronger average predictive performance. SHAP values were calculated only for held-out observations within the cross-validation procedure and were then aggregated across folds. Mean absolute SHAP values were used to summarize predictor importance, while the sign and distribution of SHAP values were used to describe whether predictor values tended to contribute to higher or lower predicted HGAI. SHAP values were interpreted as model-based predictive contributions and not as causal effects. The SHAP framework follows Lundberg and Lee (2017), with tree-based interpretation informed by Lundberg et al. (2020).

The SHAP explanation model was expressed as:

(10) $$g\left(x^{\prime }\right)=\phi_{0}+\sum_{j=1}^{M}\phi_{j}{x^{\prime }}_{j}$$

where *g*(*x*′) is the local explanation, *φ*_0_ is the baseline prediction, *φ_j_* is the SHAP contribution of predictor *j*, and *M* is the number of predictors.

Several sensitivity analyses were conducted. First, responsible AI disclosure was added to the predictor set to quantify the incremental predictive information associated with this closely related construct. Second, AI-use frequency was added as a covariate, and the models were estimated separately among respondents reporting at least moderate AI use and among those reporting high or very high AI use. These analyses distinguished concealment-related prediction from simple differences in the frequency of generative AI use.

Binary classification was retained only as a secondary threshold-sensitivity analysis. HGAI cutoffs of 2.50, 3.00, and 3.50 were examined using logistic regression, random forest, and XGBoost. Classification performance was evaluated using ROC-AUC, precision-recall AUC, accuracy, precision, recall, specificity, F1 score, balanced accuracy, Matthews correlation coefficient, and the Brier score. Calibration intercepts and slopes were also calculated from out-of-fold probabilities. The 3.00 cutoff was treated as a scale-midpoint specification rather than as a validated diagnostic threshold.

For the binary sensitivity models, class probability was represented by the logistic function:

(11) $$P\left(y_{i}=1|x_{i}\right)=\frac{1}{1+exp\left(-z_{i}\right)}$$

Classification balance was also summarized using the F1 score:

(12) $$F_{1}=2\;\frac{Precision\times Recall}{Precision+Recall}$$

### 4.3. Exploratory Evidence-Informed Choquet Intervention Prioritization

The decision-support stage used the Choquet Integral to explore how managerial interventions could be prioritized when structural importance, predictive importance, and interactions among criteria are considered jointly. This stage was designed as an exploratory prioritization exercise rather than as an empirical test of intervention effectiveness. No expert panel or field intervention evaluation was used; therefore, the resulting rankings represent priorities under explicit analytical and scenario assumptions rather than causal evidence about which intervention would produce the largest real-world effect.

Nine upstream criteria were included: AI disclosure anxiety (AIDA), competence penalty fear (CPF), AI policy clarity (AIPC), social intelligence (SI), official AI tool adequacy (OAIT), psychological safety (PS), AI metacognition (AIM), human–AI trust (HAIT), and emotional intelligence (EI). Responsible AI disclosure was not used as an intervention criterion because it is an endogenous behavioral outcome and is conceptually close to hidden generative AI use.

Eight managerial alternatives were considered: clear AI disclosure policy, a non-punitive AI-use disclosure system, psychological safety training, managerial AI literacy training, official secure AI tools, responsible AI-use guidelines, AI metacognition and verification training, and a client transparency protocol.

The criterion set was defined as:

(13) $$C=\{c_{1},c_{2},\ldots ,c_{m}\}$$

where *C* denotes the complete criterion set and *m* = 9 in the present analysis.

The intervention alternatives were represented as:

(14) $$A=\{a_{1},a_{2},\ldots ,a_{s}\}$$

where *A* denotes the set of intervention alternatives and *s* = 8.

The intervention-performance matrix was expressed as:

(15) $$X={\left[x_{ij}\right]}_{s\times m}$$

where *x_ij_* is the scenario score assigned to alternative *a_i_* for criterion *c_j_*.

For benefit-type criteria, normalization was calculated as:

(16) $$r_{ij}=\frac{x_{ij}}{\underset{i}{max}\left(x_{ij}\right)}$$

For cost-type criteria, normalization was calculated as:

(17) $$r_{ij}=\frac{\underset{i}{min}\left(x_{ij}\right)}{x_{ij}}$$

where *r_ij_* is the normalized performance value of alternative *a_i_* under criterion *c_j_*.

Criterion importance combined structural and predictive evidence. The SEM component reflected each criterion’s total standardized association with HGAI, including relevant indirect pathways through intermediate constructs. The predictive component was derived from mean absolute out-of-fold SHAP importance in the primary RAID-excluded XGBoost model. Both components were normalized to sum to one. Their combination was controlled by the parameter *λ*, such that *w_j_*(*λ*) = *λw*_j, SEM_ + (1 − *λ*)*w*_j_, _SHAP_. The baseline specification used *λ* = 0.50, while *λ* = 0, 0.25, 0.75, and 1.00 were examined in sensitivity analysis. Thus, equal weighting served as one transparent baseline assumption rather than as an untested requirement of the method.

The SEM-based and SHAP-based importance components were normalized separately:

(18) $${SEM}_{j}^{norm}=\frac{{SEM}_{j}}{\sum_{j=1}^{m}{SEM}_{j}}$$

(19) $${SHAP}_{j}^{norm}=\frac{{SHAP}_{j}}{\sum_{j=1}^{m}{SHAP}_{j}}$$

The two evidence sources were then combined through the λ-weighting function:

(20) $$w_{j}\left(\lambda \right)=\lambda w_{j,SEM}+\left(1-\lambda \right)w_{j,SHAP}$$

where *λ* ∈ [0, 1]. The baseline specification used *λ* = 0.50, while *λ* = 0, 0.25, 0.50, 0.75, and 1.00 were evaluated in sensitivity analysis.

Intervention performance was represented using a five-level scenario rubric: 0.00 = no clear target, 0.25 = weak secondary relevance, 0.50 = moderate relevance, 0.75 = strong proximal relevance, and 1.00 = direct target. The rubric was applied consistently across all intervention-criterion combinations based on the theoretical mechanisms represented by each intervention. These values are structured scenario scores and should not be interpreted as observed intervention-effectiveness estimates.

The intervention-performance values were not obtained by normalizing observed intervention outcomes or by eliciting expert judgments. They represent theory-informed scenario mappings based on the conceptual mechanisms developed in Section 2.3, Section 2.4, Section 2.5 and Section 2.6. A score of 1.00 was assigned when an intervention directly targeted the criterion; 0.75 when the criterion represented a strong proximal target; 0.50 when the expected connection was moderate or indirect; 0.25 when the intervention had only secondary relevance; and 0.00 when no clear conceptual targeting relationship was specified. The same decision rule was applied to all intervention–criterion combinations before Choquet aggregation. The normalization equations apply to the resulting decision matrix and should not be interpreted as the source of the original scenario scores.

To represent non-additive relationships among criteria, a positive 2-additive Choquet capacity was constructed. Singleton capacity mass reflected the combined SEM-SHAP criterion importance, while pairwise interaction mass was allocated in proportion to out-of-fold SHAP interaction strengths. Singleton and pairwise Möbius coefficients were constrained to be nonnegative and normalized so that total capacity mass equaled one, preserving the boundary and monotonicity properties required for a valid capacity. For an intervention profile *x*, the score was represented as *C*(*x*) = Σ*_i_ m_i_*x*_i_* + Σ*_i_*<*_j_ m_ij_* min(*x_i_*, *x_j_*), where *m_i_* denotes singleton importance and *m_ij_* denotes the pairwise interaction contribution. The use of the Choquet integral for non-additive multicriteria aggregation follows established formulations in Grabisch (1996) and Grabisch and Labreuche (2010).

The Choquet capacity was defined on the power set of C as:

(21) $$\mu :2^{C}\to \left[0,1\right]$$

with the boundary conditions:

(22) $$\mu \left(⌀\right)=0,\;\;\mu \left(C\right)=1$$

and the monotonicity condition:

(23) $$S\subseteq T\Rightarrow \mu \left(S\right)\leq \mu \left(T\right)$$

For each intervention, normalized criterion values were ordered as:

(24) $$r_{i|1}\leq r_{i|2}\leq \cdots \leq r_{i|m}$$

The Choquet Integral score was then calculated as:

(25) $$CI\left(a_{i}\right)=\sum_{j=1}^{m}\left(r_{i|j}-r_{i|j-1}\right)\mu \left(A_{j}\right)$$

where *μ*(*A_j_*) is the capacity of the subset of criteria from position *j* through *m* and *r_i_*_|0_ = 0.

The alternatives were ranked in descending order of their Choquet scores. The highest-scoring alternative was interpreted as the highest-priority option under the specified evidence weights, scenario scores, and interaction structure. The ranking therefore supports comparative decision making under stated assumptions but does not establish that the leading alternative is causally more effective than the remaining interventions.

(26) $$Rank\left(a_{i}\right)=descending\;\left[CI\left(a_{i}\right)\right]$$

The highest score therefore indicates the highest-priority alternative under the specified evidence weights, scenario scores, and interaction assumptions, not proven intervention effectiveness.

### 4.4. Sensitivity and Robustness Analysis

The stability of the intervention ranking was evaluated under multiple forms of uncertainty rather than relying on a single weight perturbation. First, the balance between structural and predictive evidence was varied across λ values from 0 to 1. Second, the additive weighted model was compared with the Choquet specification to determine whether pairwise interaction modeling materially changed the ordering of alternatives. Third, the interaction-capacity contribution was perturbed to assess sensitivity to the estimated strength of criterion interactions.

Uncertainty in the intervention-performance matrix was examined through Monte Carlo simulation. In the moderate-uncertainty analysis, λ varied continuously over its feasible range, interaction capacity was perturbed around its empirical estimate, and intervention-performance scores were allowed to vary by up to ±0.10. A stronger stress test allowed each performance score to vary by up to ±0.25, corresponding to one full category of the scenario rubric. For each simulation, Choquet scores and ranks were recalculated.

Rank robustness was summarized using Spearman rank correlations with the baseline ordering, the probability that each alternative ranked first, and the probability that each alternative appeared among the top three. Rank-reversal checks were also examined across alternative λ specifications and perturbation scenarios. These analyses were used to distinguish conclusions that remained stable across plausible assumptions from rankings that were more sensitive to modeling choices.

Spearman rank correlation was calculated as:

(27) $$\rho =1-\frac{6\sum_{i=1}^{n}d_{i}^{2}}{n\left(n^{2}-1\right)}$$

where *d_i_* is the difference between the baseline and sensitivity-scenario rank of alternative *i* and *n* is the number of alternatives.

### 4.5. Computational Environment

The analyses did not require specialized high-performance computing infrastructure or GPU acceleration. PLS-SEM analyses were conducted using SmartPLS version 4.1.1.7, and preliminary data screening and descriptive analyses were performed using IBM SPSS Statistics version 31.0.1.0. The predictive and decision-support analyses were implemented in Python version 3.14.3, using XGBoost version 3.2.0, scikit-learn version 1.8.0, SHAP version 0.50.0, pandas version 3.0.1, and NumPy version 2.4.2. Fixed random seeds were used for cross-validation partitioning and stochastic model estimation to support reproducibility. Given the sample size and analytical procedures, the computations can be performed on a standard contemporary desktop or laptop computer.

## 5. Results

### 5.1. Descriptive Statistics and Correlations

Descriptive analyses were conducted for the final sample of 564 consulting and professional service employees in Türkiye. The demographic profile is presented in Table 3.

AI-use characteristics are reported in Table 4. All respondents met the prior-use eligibility criterion; the frequency categories therefore represent variation among GenAI users rather than a contrast between users and non-users.

Table 5 presents the means, standard deviations, and correlations among the main study variables.

### 5.2. Measurement Model Results

The measurement model was evaluated using covariance-based CFA alongside the PLS-based measurement assessment. Table 6 reports global CFA fit and supplementary common-method diagnostics.

The 11-factor CFA showed excellent global fit, χ^2^(1484) = 1583.17, CFI = 0.992, TLI = 0.991, RMSEA = 0.011, and SRMR = 0.032, whereas the single-factor model fit poorly. Harman’s first unrotated factor accounted for 19.4% of item variance, and full-collinearity VIFs ranged from 1.232 to 1.670. These diagnostics do not eliminate the possibility of common-method variance, but they provide no indication that a single common factor dominated the covariance structure.

Discriminant validity was evaluated using HTMT values. As shown in Table 7, all HTMT values were below 0.85. The RAID–HGAI HTMT value was 0.464, and the dedicated two-factor RAID–HGAI CFA showed excellent fit, whereas a one-factor specification fit poorly. These results support the treatment of responsible AI disclosure and hidden generative AI use as related but empirically distinct constructs.

Taken together, the CFA, reliability, HTMT, and RAID–HGAI comparison results support the 11-construct measurement structure. Several CFA-based AVE values were marginally below 0.50, so convergent validity is interpreted cautiously for those constructs rather than treated as uniformly strong.

### 5.3. Structural Model and Robustness Results

Table 8 compares the PLS-SEM standardized coefficients with the covariance-based SEM estimates. All 14 hypothesized relationships retained the same direction across estimators, and the PLS bootstrap confidence intervals excluded zero for each hypothesized path. The covariance-based structural model demonstrated good global fit, χ^2^(1510) = 1698.66, CFI = 0.984, TLI = 0.983, RMSEA = 0.015, and SRMR = 0.045. The similarity in coefficient direction across PLS-SEM and CB-SEM indicates that the substantive pattern of the structural relationships was not dependent on a single estimator.

Additional robustness checks produced the same general interpretation. Structural VIF values ranged from approximately 1.00 to 1.31, indicating no problematic multicollinearity. The exploratory AIM × HAIT interaction for responsible AI disclosure was not significant (β = −0.047, *p* = .169; ΔR^2^ ≈ 0.002), whereas the indirect AIM → HAIT → RAID association was positive and its bootstrap confidence interval excluded zero (βind ≈ 0.052, 95% CI [0.028, 0.080]). Thus, human–AI trust appears more relevant as part of the disclosure pathway than as a moderator of the AI metacognition relationship.

The core structural coefficients also remained stable when AI familiarity, AI-use frequency, formal AI policy, responsible-AI training, and client-communication involvement were included as controls. Multi-group comparisons based on client-communication involvement (*n* = 316 versus *n* = 248) produced no statistically significant differences across the 14 structural paths. The professional-area comparison between support-oriented IT/HR roles (*n* = 152) and client/advisory-oriented roles (*n* = 389), with 23 “Other” cases excluded from this comparison, likewise produced no path difference at *p* < .05, providing no evidence that the structural pattern was driven by a single occupational subgroup. These additional structural robustness checks are summarized in Supplementary Table S5.

The explanatory power and predictive relevance of the structural model are summarized in Table 9. PLS-SEM explained 39.2% of HGAI variance and 34.3% of RAID variance; the corresponding latent CB-SEM R^2^ values were 0.471 and 0.440.

Effect sizes are presented in Table 10.

The structural findings are interpreted as associations rather than causal effects because the study is cross-sectional. Alternative reverse-direction specifications were statistically plausible, including significant paths from HGAI to RAID, AIDA, and CPF. These checks do not establish that the reverse specifications are theoretically preferable; rather, they reinforce that temporal and causal ordering cannot be determined from one-wave observational data.

**Figure 2:**
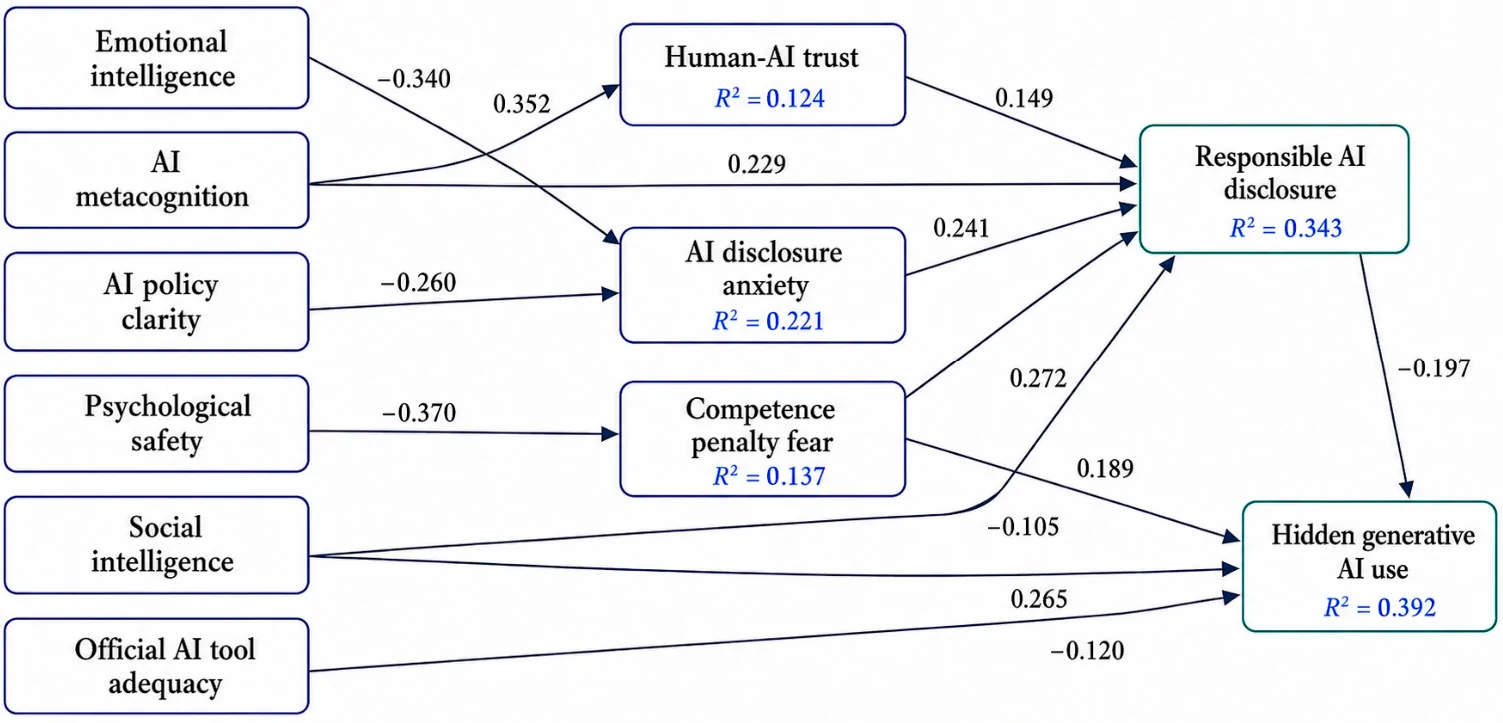
Structural model results with CB-SEM robustness evidence.

Figure 2 presents the PLS-SEM structural coefficients and endogenous R^2^ values; the CB-SEM global fit and coefficient robustness are reported in Table 8.

### 5.4. Predictive Modeling and Out-of-Fold SHAP Results

Continuous HGAI was used as the primary predictive outcome to preserve information in the six-item construct. RAID was excluded from the primary predictor set because of its conceptual proximity to concealment; RAID-inclusive prediction was treated as a sensitivity analysis.

Cross-validated model performance is reported in Table 11. Ridge regression produced the strongest average predictive performance, while XGBoost remained useful as an interpretable nonlinear comparator.

The predictive results do not indicate an advantage of XGBoost over the simpler regularized linear model. Ridge regression achieved the strongest average cross-validated performance (MAE = 0.422, RMSE = 0.521, mean R^2^ = 0.341), whereas XGBoost achieved MAE = 0.431, RMSE = 0.536, and mean R^2^ = 0.301 in the repeated cross-validation comparison. XGBoost was therefore retained primarily for nonlinear interpretation through out-of-fold SHAP values rather than treated as the best-performing predictor.

Including responsible AI disclosure as an additional predictor modestly improved out-of-fold performance (Ridge R^2^ = 0.369; XGBoost R^2^ = 0.352), and RAID became the third-largest XGBoost SHAP contributor.Because RAID is conceptually close to concealment and is an endogenous construct in the structural model, this specification is treated as a sensitivity analysis rather than the primary predictive model. The RAID-added and AI-use-frequency restricted-sample results are reported in Supplementary Table S6.

Binary HGAI classifications were retained only as sensitivity analyses. Table 12 shows that class balance, recall, precision, and calibration vary materially across thresholds, supporting the use of continuous HGAI as the primary outcome. At the 3.00 threshold, the out-of-fold XGBoost classifier achieved AUC = 0.753 and recall = 0.420; these results indicate moderate discrimination but do not support interpretation of the model as a screening instrument.

SHAP values were calculated only for held-out outer-fold observations. The resulting out-of-fold predictor-importance ranking is reported in Table 13.

**Figure 3:**
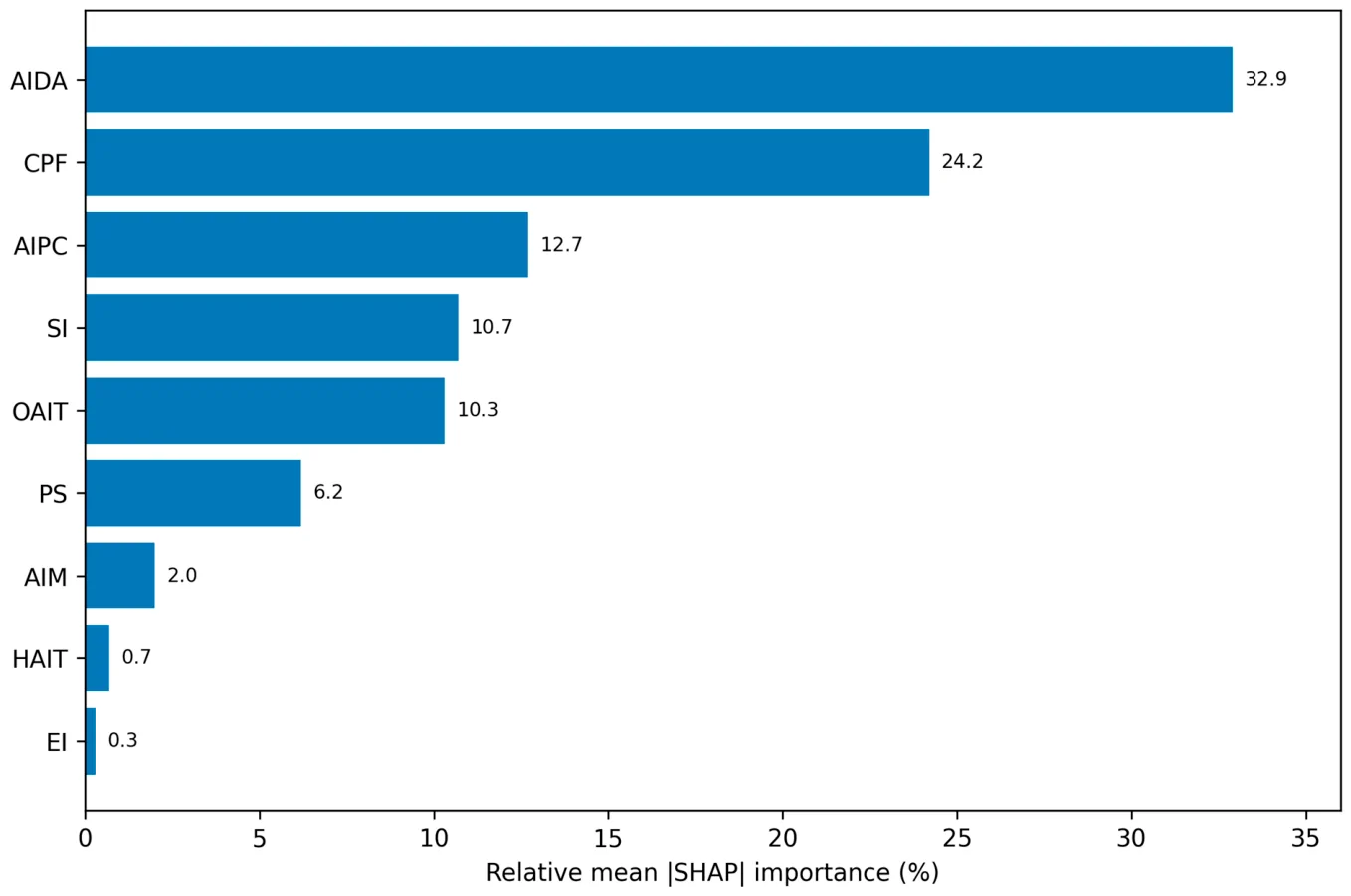
Out-of-fold SHAP importance for continuous hidden generative AI use.

Figure 3 presents the out-of-fold SHAP importance profile for the RAID-excluded primary XGBoost model.

AI disclosure anxiety and competence penalty fear were the two dominant predictive contributors, accounting for 32.9% and 24.2% of total mean absolute SHAP importance, respectively. AI policy clarity, social intelligence, and official AI tool adequacy formed a second predictive tier. The sign patterns of the out-of-fold SHAP values indicated that higher AIDA and CPF generally contributed to higher predicted HGAI values, whereas higher AIPC, SI, OAIT, and PS generally contributed to lower predicted values.

Human–AI trust showed very low incremental importance for direct HGAI prediction. This does not conflict with its significant structural association with responsible disclosure because the SEM and SHAP analyses address different outcomes and different forms of importance.

Sensitivity analyses that added AI-use frequency or restricted the sample to moderate-or-higher and high-or-higher GenAI users retained meaningful predictive performance. For the moderate-or-higher subgroup (*n* = 442), Ridge regression yielded out-of-fold R^2^ = 0.330, while the high-or-higher subgroup (*n* = 226) yielded R^2^ = 0.377. The predictive signal therefore remained evident after excluding lower-frequency users.

### 5.5. Exploratory Evidence-Informed Choquet Intervention Prioritization

The intervention stage was specified as exploratory evidence-informed decision support. RAID was excluded from the criterion set, SEM criterion importance was based on total structural pathways to HGAI, and predictive importance was derived from RAID-excluded out-of-fold SHAP values. The baseline λ = 0.50 specification gives equal emphasis to structural and predictive evidence, while alternative λ values are examined in sensitivity analysis.

The resulting evidence-informed criterion weights are shown in Table 14. AI disclosure anxiety and competence penalty fear receive the largest baseline weights. Pairwise out-of-fold SHAP interactions accounted for approximately 11.6% of the aggregate capacity mass in the 2-additive Choquet specification, allowing interaction information to refine the ranking without dominating the singleton criterion weights.

Table 15 presents the intervention-performance matrix generated using the five-level mapping rule specified in Section 4.3. The values represent theory-informed scenario assumptions rather than empirical estimates of intervention effectiveness. The matrix is therefore used for exploratory decision support and is examined under alternative weighting and score-perturbation assumptions in the sensitivity analyses.

The baseline Choquet ranking is presented in Table 16. A2, the non-punitive AI-use disclosure system, receives the highest priority score under the specified assumptions.

**Figure 4:**
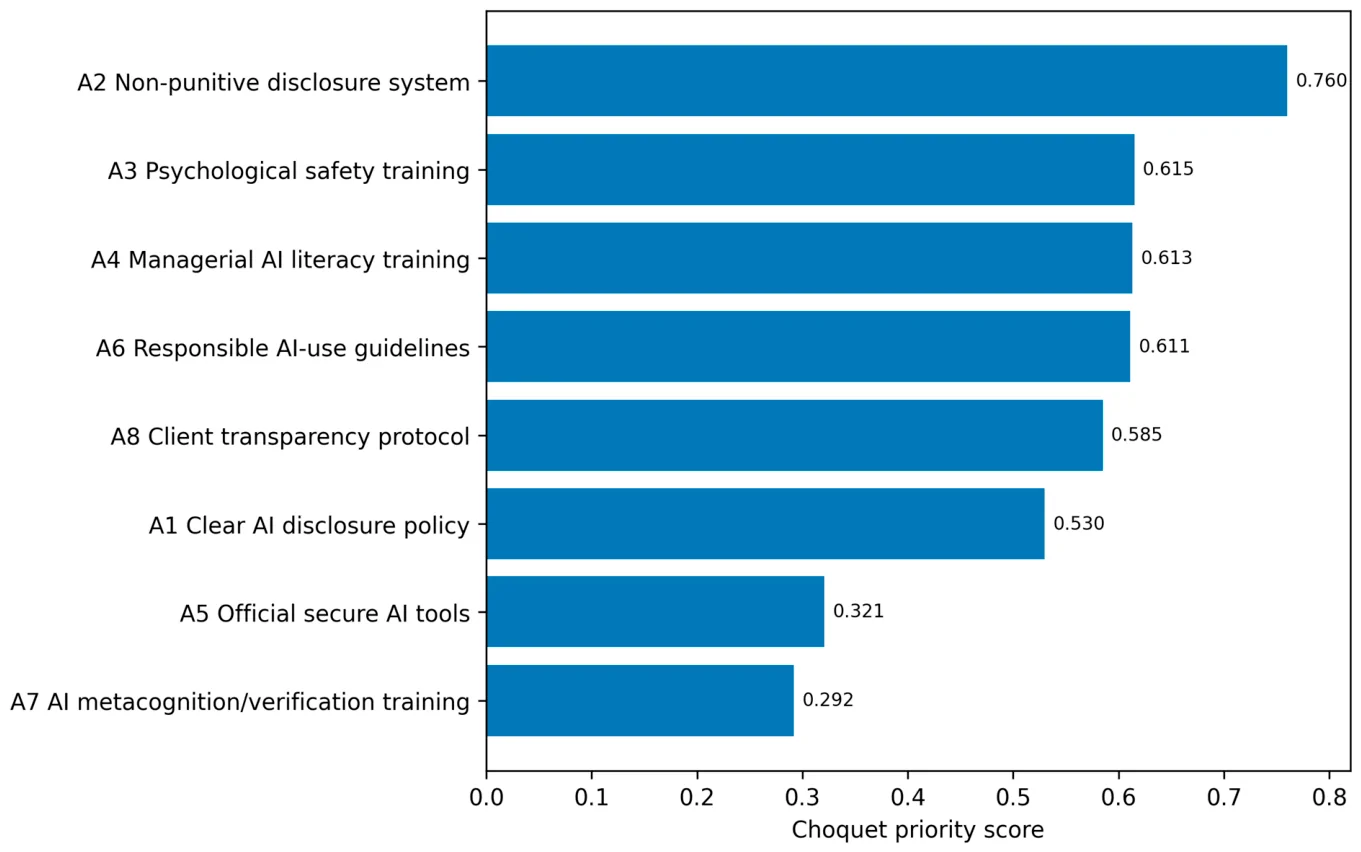
Choquet-Based Prioritization of Managerial Interventions.

Figure 4 presents the Choquet priority scores.

A2 is interpreted as the most consistently prioritized intervention under the modeled assumptions, not as an empirically proven most effective intervention.

The middle ordering is more sensitive: psychological safety training, managerial AI literacy training, and responsible AI-use guidelines occupy the next tier, and their precise order depends on the balance between structural and predictive evidence and on interaction assumptions.

### 5.6. Sensitivity and Robustness of Intervention Rankings

Weighting sensitivity was examined by varying λ from 0 (prediction-only weighting) to 1 (SEM-only weighting). The resulting rankings are reported in Table 17.

A2 remained first across every λ scenario, while the relative ordering of A3, A4, and A6 varied. This pattern distinguishes a stable leading alternative from a less certain middle ranking.

Monte Carlo rank acceptability was additionally evaluated under uncertainty in λ, interaction capacity, and intervention-performance scores. The results are summarized in Table 18.

The Monte Carlo results show that A2 remained the leading alternative in all simulations under moderate uncertainty and in 85.1% of simulations when each intervention score was allowed to vary by a full rubric category (±0.25).

The analysis therefore supports robustness of the leading priority but not complete certainty about the ordering of the middle-ranked interventions.

Finally, Table 19 compares the additive weighted ranking with the data-informed Choquet ranking. Modeling pairwise interactions changes the middle ordering but does not create the leading A2 result.

The ranking evidence is interpreted as exploratory scenario-based decision support. It should not be interpreted as causal evidence that any intervention will produce a specific behavioral effect.

## 6. Discussion and Implications

The findings indicate that hidden generative AI use in consulting and professional services is associated with a broader combination of social, emotional, cognitive, and organizational conditions rather than representing only an ethical or compliance-related issue. Employees may be reluctant to disclose AI assistance when doing so is perceived as professionally risky, particularly in work settings where expertise, originality, analytical judgment, and client trust are central to professional evaluation. In this context, concealment may reflect concerns about how AI-supported work will be interpreted by supervisors, colleagues, or clients rather than an intention to mislead others. This interpretation is consistent with the employee silence literature, which suggests that employees may withhold relevant information when expressing it is perceived as risky (Morrison & Milliken, 2000; Milliken et al., 2003; Van Dyne et al., 2003). Hidden AI use extends this logic to human–AI collaboration because the withheld information concerns the process through which professional work was produced.

AI disclosure anxiety and competence penalty fear showed particularly important relationships with hidden generative AI use. Both constructs were positively associated with concealment in the structural model and emerged as the two strongest predictors in the out-of-fold SHAP analysis. These findings are consistent with fear-of-negative-evaluation and impression-management perspectives. Leary (1983) explains that individuals may experience anxiety when they anticipate unfavorable evaluation, while Bolino and Turnley (1999) emphasize that employees actively manage impressions of their competence and professionalism. Applied to AI-supported consulting, employees may be reluctant to acknowledge meaningful AI assistance when they believe that disclosure could diminish the perceived originality, expertise, or value of their contribution. The prominence of competence penalty fear also corresponds with research on AI and professional identity, which suggests that intelligent technologies may create tensions when employees perceive them as challenging the symbolic value of human expertise (Mirbabaie et al., 2022; Bankins & Formosa, 2023).

Responsible AI disclosure was negatively associated with hidden generative AI use, but the two constructs should not be interpreted as exact opposites. The measurement analyses supported their empirical distinctiveness, indicating that affirmative transparency and concealment represent related but separate behaviors. Employees may disclose AI assistance in some professional situations while remaining reluctant to do so in others depending on the task, audience, or perceived disclosure expectations. This distinction contributes to responsible AI research by extending transparency from the technical characteristics of AI systems to the behavior of employees who use them. Prior research emphasizes transparency, accountability, and explainability as foundations of trustworthy AI use (Barredo Arrieta et al., 2020; Rai, 2020; Meske et al., 2022). In professional practice, responsible disclosure similarly involves communicating when AI made a substantive contribution, how its output was evaluated, and where human judgment remained central.

AI metacognition was positively associated with responsible AI disclosure and also showed an indirect association with disclosure through human–AI trust. Employees with greater awareness of AI capabilities, limitations, and risks may be better positioned to recognize when an AI contribution is sufficiently meaningful to require transparency. This interpretation is consistent with AI literacy research emphasizing the ability to evaluate and critically use AI rather than simply operate it (Long & Magerko, 2020; Ng et al., 2021; Wang et al., 2023).

Human–AI trust was also positively associated with responsible disclosure, although its direct predictive importance for hidden AI use was small in the SHAP analysis. This difference is theoretically informative rather than contradictory: the structural model positions human–AI trust primarily within the disclosure pathway, whereas the predictive analysis assesses its unique contribution to the direct prediction of hidden AI use. The nonsignificant AIM × HAIT interaction further suggests that the relationship is better understood through the indirect AIM → HAIT → responsible disclosure pathway than through moderation.

The organizational findings further emphasize the importance of the context in which AI-supported work occurs. Psychological safety was negatively associated with competence penalty fear, while AI policy clarity was negatively associated with disclosure anxiety and positively associated with responsible disclosure. These patterns are consistent with Edmondson’s (1999) work on psychological safety and with research emphasizing supportive leadership, interpersonal trust, and open communication (O’Donovan & McAuliffe, 2020). In AI-supported consulting work, psychological safety may make it easier for employees to discuss how AI contributed to a task without assuming that such disclosure will automatically undermine perceptions of competence. Similarly, clear policies may reduce ambiguity surrounding acceptable AI use, disclosure requirements, data handling, and the use of client information.

Official AI tool adequacy was negatively associated with hidden generative AI use and positively associated with responsible disclosure. This finding suggests that governance cannot depend solely on formal rules. Employees also require approved, secure, and task-relevant tools that make responsible AI use practically feasible. Where such tools are inadequate, employees may be more inclined to rely on unofficial alternatives and may subsequently be less comfortable communicating their use. This interpretation is consistent with research emphasizing organizational readiness, technological infrastructure, and work design as important conditions of responsible AI adoption (Jöhnk et al., 2021; Robert et al., 2020; Sowa et al., 2021). Accordingly, effective workplace AI governance appears to require both behavioral expectations and the resources necessary to follow them.

The predictive analysis provides complementary evidence. Ridge regression achieved the strongest average cross-validated performance, indicating that much of the predictive structure can be captured without relying on a highly complex nonlinear model. XGBoost nevertheless remained useful as an interpretable nonlinear model through out-of-fold SHAP analysis. AI disclosure anxiety and competence penalty fear accounted for the largest shares of predictive importance, followed by AI policy clarity, social intelligence, and official AI tool adequacy. This convergence between structural and predictive evidence strengthens the interpretation that perceived evaluation risk and organizational conditions are central to understanding variation in hidden AI use. At the same time, the results underscore the importance of distinguishing explanatory associations from predictive importance: a statistically meaningful structural relationship does not necessarily imply high incremental predictive contribution.

The predictive sensitivity analyses also address the possibility that hidden AI use simply reflects how frequently employees use generative AI. All respondents had prior work-related GenAI experience, and predictive performance remained meaningful when the analyses were restricted to respondents reporting at least moderate AI use and to those reporting high or very high AI use. The primary analysis therefore treats HGAI as a continuous behavioral measure rather than equating low AI-use frequency with low concealment risk. Binary classifications based on alternative HGAI thresholds produced materially different class balances and sensitivity levels, further supporting the use of continuous HGAI as the principal predictive outcome rather than interpreting any single threshold as a validated risk boundary.

The exploratory Choquet analysis adds a decision-support perspective by examining how the structural and predictive evidence may inform intervention prioritization under explicit assumptions. Under the baseline scenario, the non-punitive AI-use disclosure system received the highest priority score, followed by psychological safety training and managerial AI literacy training, with responsible AI-use guidelines also remaining among the higher-ranked alternatives. Importantly, these rankings do not represent observed intervention effectiveness. Criterion importance was informed by structural and predictive evidence, whereas intervention-performance values were assigned through a transparent scenario-scoring rubric. The analysis should therefore be interpreted as an exploratory prioritization exercise rather than as evidence that one intervention will causally produce the greatest change in hidden AI use. The stability analyses nevertheless showed that the non-punitive disclosure system remained the highest-ranked alternative across different structural-predictive weighting assumptions and under substantial perturbation of the intervention scores.

The theoretical contribution of the study lies first in conceptualizing hidden generative AI use as a social-cognitive and human–AI interaction phenomenon rather than exclusively as misconduct or policy non-compliance. The findings connect concealment with professional-image concerns, evaluation anxiety, competence-related perceptions, organizational safety, and AI governance conditions. Second, the study distinguishes responsible AI disclosure from hidden AI use conceptually and empirically, allowing affirmative transparency and concealment to be examined as related but distinct workplace behaviors. Third, the findings connect social intelligence, emotional intelligence, AI metacognition, and human–AI trust with disclosure-related processes, extending human–AI interaction research beyond technology acceptance toward questions of professional transparency and accountability.

The study also contributes to workplace AI governance by highlighting the joint relevance of policy clarity, adequate official tools, psychological safety, and employees’ perceptions of professional evaluation. This perspective is particularly relevant in consulting and professional services, where AI-supported outputs may be incorporated into reports, recommendations, presentations, and client communication. The analytical framework further combines structural modeling, predictive model comparison, explainable machine learning, and exploratory intervention prioritization. These components serve different purposes: SEM evaluates theoretically specified relationships, predictive models evaluate out-of-sample performance, SHAP helps interpret nonlinear prediction, and Choquet analysis provides scenario-based decision support. Their integration therefore allows explanatory, predictive, and managerial perspectives to be considered together without treating them as methodologically equivalent.

From a practical perspective, consulting and professional-service organizations should establish clear expectations concerning when substantive AI assistance should be disclosed and how such disclosure should be documented, particularly for client-facing outputs. Equally important, organizations should avoid creating a climate in which responsible disclosure is automatically associated with poor performance, reduced expertise, or misconduct. The consistently high priority assigned to a non-punitive disclosure system suggests that governance approaches should combine transparency expectations with credible assurance that responsible disclosure will be evaluated fairly. Psychological safety initiatives and managerial AI literacy may further help employees discuss AI-supported work without unnecessary professional-image concerns.

Organizations should also provide secure and task-appropriate official AI tools and strengthen AI metacognition and verification capabilities. Training should focus not only on how to operate generative AI but also on how to identify unreliable outputs, verify evidence, protect confidential information, distinguish minor technical assistance from substantive AI contribution, and determine when transparency is appropriate. Client-facing AI transparency protocols may further help employees communicate AI-supported work consistently without suggesting that human professional responsibility has been transferred to the technology. Managers, in turn, should evaluate AI-supported outputs based on the quality of verification, protection of client information, appropriateness of AI use, and the professional judgment added by the employee.

The findings suggest that workplace AI governance should not frame responsible AI use as a choice between human expertise and technological assistance. In knowledge-intensive professional work, expertise increasingly includes the ability to determine when AI is appropriate, critically evaluate its outputs, remain accountable for resulting decisions, and communicate substantive AI assistance transparently. Creating such conditions may require a combination of clear policies, adequate technological resources, psychologically safe disclosure practices, calibrated human–AI trust, and managerial expectations that recognize responsible AI use as part of contemporary professional judgment.

The increasing availability of generative AI changes how organizations may need to evaluate professional outputs. When AI can substantially improve the fluency, structure, and visual polish of reports, proposals, presentations, and other documents, surface quality alone provides less information about the employee’s independent reasoning or professional contribution. Evaluation should therefore place greater emphasis on the quality and traceability of the underlying evidence, the appropriateness of the analytical process, the verification of AI-supported content, and the human judgment used to reach the final conclusion.

For consulting reports and other consequential client-facing outputs, organizations can adopt proportionate documentation requirements that distinguish minor technical assistance from substantive AI contribution. Where generative AI materially contributes to analysis, synthesis, drafting, or the development of recommendations, employees can be asked to identify the role of AI and retain an appropriate record of key sources, assumptions, calculations, verification steps, and material changes made through human review. Managers and clients should evaluate whether claims are supported by credible evidence, whether factual statements and quantitative outputs have been checked, and whether the responsible professional can explain why the final recommendation is appropriate. Such practices shift attention from whether AI was used toward whether the resulting work is transparent, verifiable, and professionally accountable.

Interactive evaluation can provide an additional safeguard when the authenticity or depth of an AI-assisted output is important. Short oral explanations, follow-up questions, scenario discussions, or requests to defend key assumptions can help assess whether an employee understands the material, can interpret the evidence, and can exercise judgment beyond the generated text. This approach is particularly relevant for high-stakes recommendations because responsibility should remain with the professional who submits or endorses the work rather than with the generative AI system.

A similar principle applies to résumés, CVs, cover letters, and other documents used to evaluate professional qualifications. Because generative AI can improve wording and presentation, polished language should not be treated as sufficient evidence of competence. Organizations can instead verify substantive claims through credential and employment checks, references, work samples, structured interviews, task-based assessments, or demonstrations of relevant knowledge and skills. The appropriate focus is therefore the accuracy of the claims and the applicant’s demonstrated capability rather than the mere use of AI for language support.

These practices also suggest that disclosure requirements should be calibrated to the materiality of AI assistance. Requiring disclosure for every spelling, grammar, or formatting suggestion could create unnecessary administrative burden, whereas substantive AI involvement in analysis, interpretation, or recommendation development may warrant clearer transparency. A materiality-based approach can preserve the benefits of generative AI while strengthening evidence quality, traceability, and human accountability in professional evaluation.

## 7. Conclusions

This study examined hidden generative AI use among consulting and professional-service employees in Türkiye and focused on why employees may use generative AI for work-related tasks while remaining reluctant to disclose meaningful AI assistance. The findings indicate that hidden AI use is associated with social, emotional, cognitive, and organizational conditions rather than representing only an ethical or compliance issue. AI disclosure anxiety and competence penalty fear showed the strongest positive associations with concealment and were also the most important predictors in the out-of-fold SHAP analysis. Responsible AI disclosure, social intelligence, official AI tool adequacy, policy clarity, psychological safety, AI metacognition, and human–AI trust were also relevant to disclosure-related behavior. Importantly, AI-use frequency and concealment were treated as conceptually distinct behaviors.

The study contributes by conceptualizing hidden generative AI use as a social-cognitive and human–AI interaction phenomenon, distinguishing responsible AI disclosure from concealment, and extending workplace AI governance research to consulting and professional services. Methodologically, PLS-SEM was combined with CB-SEM robustness analysis, cross-validated predictive modeling, out-of-fold SHAP interpretation, and exploratory Choquet intervention prioritization. Ridge regression achieved the strongest average predictive performance, while XGBoost was used to examine nonlinear predictive importance. The Choquet analysis identified a non-punitive AI-use disclosure system as the most consistently prioritized intervention under the modeled assumptions, followed in the baseline scenario by psychological safety training and managerial AI literacy training. These rankings represent exploratory decision support rather than evidence of causal intervention effectiveness.

Several limitations should be acknowledged. The sample was restricted to consulting and professional-service employees in Türkiye, which limits generalizability. The cross-sectional design does not establish temporal or causal ordering, and the use of self-reported data may introduce common-method and social-desirability concerns. The study also did not directly observe AI-use behavior or disclosure practices. Because survey-start information and characteristics of nonrespondents were unavailable, nonresponse bias could not be assessed directly. Although the measurement model demonstrated good overall fit and discriminant validity, several CFA-based AVE values were marginally below 0.50. In addition, the intervention-performance scores used in the Choquet analysis were structured scenario assumptions rather than observed intervention outcomes.

Future studies could use longitudinal, experimental, multi-source, and cross-country designs to examine how AI disclosure behavior develops over time and across institutional settings. Behavioral measures, AI-use logs, audit trails, and observed disclosure decisions could complement self-report measures where ethically and legally appropriate. Further research could also examine leadership, client pressure, workload, AI governance maturity, and professional identity climate, as well as compare alternative explainable machine learning and decision-support methods.

Responsible generative AI use in professional services depends not only on access to technology but also on clear disclosure expectations, suitable official tools, critical evaluation of AI outputs, psychological safety, and fair managerial responses to AI-supported work. Creating conditions in which employees can communicate meaningful AI assistance without unnecessary professional-image risk may therefore be an important part of responsible workplace AI governance.

## Figures and Tables

**Table 1:** Measurement structure.

Construct	Code	Items	Source/Basis
Social intelligence	SI	5	Silvera et al. (2001), adapted
Emotional intelligence	EI	5	Wong and Law (2017), adapted
AI metacognition	AIM	6	Schraw and Dennison (1994); Ng et al. (2021), adapted
Psychological safety	PS	5	Edmondson (1999), adapted
Human–AI trust	HAIT	5	Jian et al. (2000); Choung et al. (2023), adapted
AI policy clarity	AIPC	5	Context-adapted organizational AI policy clarity items
Official AI tool adequacy	OAIT	5	Context-adapted official AI tool adequacy items
AI disclosure anxiety	AIDA	5	Leary (1983), adapted
Competence penalty fear	CPF	5	Leary (1983); Bolino and Turnley (1999), adapted
Responsible AI disclosure	RAID	5	Van Dyne and LePine (1998); Van Dyne et al. (2003), adapted
Hidden generative AI use	HGAI	6	Morrison and Milliken (2000); Milliken et al. (2003); Van Dyne et al. (2003), adapted

**Table 2:** Measurement-model summary.

Construct	Items	α	ω/CR	CFA AVE	Std. Loading Range	Assessment
SI	5	0.827	0.828	0.492	0.643–0.766	Marginal AVE; CR adequate
EI	5	0.814	0.814	0.468	0.600–0.741	Marginal AVE; CR adequate
AIM	6	0.848	0.849	0.485	0.639–0.758	Marginal AVE; CR adequate
PS	5	0.837	0.838	0.509	0.645–0.766	AVE ≥ 0.50
HAIT	5	0.833	0.834	0.501	0.674–0.738	AVE ≥ 0.50
AIPC	5	0.834	0.836	0.506	0.638–0.807	AVE ≥ 0.50
OAIT	5	0.808	0.809	0.460	0.603–0.726	Marginal AVE; CR adequate
AIDA	5	0.817	0.818	0.475	0.645–0.779	Marginal AVE; CR adequate
CPF	5	0.798	0.799	0.445	0.599–0.714	Below 0.50; retain cautiously
RAID	5	0.839	0.841	0.514	0.680–0.767	AVE ≥ 0.50
HGAI	6	0.858	0.858	0.502	0.672–0.735	AVE ≥ 0.50

**Table 3:** Demographic profile of the sample.

Variable	Category	*n*	%
Gender	Male	269	47.7
	Female	266	47.2
	Prefer not to say	22	3.9
	Other	7	1.2
Age	18–24	30	5.3
	25–34	197	34.9
	35–44	223	39.5
	45–54	88	15.6
	55 and above	26	4.6
Education	Bachelor’s degree	247	43.8
	Master’s degree	267	47.3
	Doctoral degree	32	5.7
	Other	18	3.2
Total work experience	Less than 1 year	19	3.4
	1–3 years	81	14.4
	4–7 years	163	28.9
	8–12 years	151	26.8
	More than 12 years	150	26.6
Consulting experience	Less than 1 year	26	4.6
	1–3 years	105	18.6
	4–7 years	183	32.4
	8–12 years	151	26.8
	More than 12 years	99	17.6

**Table 4:** AI-use profile of respondents.

Variable	Category	*n*	%
AI-use frequency	1 = Very low	30	5.3
	2 = Low	92	16.3
	3 = Moderate	216	38.3
	4 = High	167	29.6
	5 = Very high	59	10.5
AI familiarity	1 = Very low	17	3.0
	2 = Low	64	11.3
	3 = Moderate	207	36.7
	4 = High	195	34.6
	5 = Very high	81	14.4
Formal AI policy	Yes	247	43.8
	No	188	33.3
	I do not know	129	22.9
Responsible AI training	Yes	213	37.8
	No	295	52.3
	I do not know	56	9.9
AI task	Report drafting	383	67.9
	Data summarization	368	65.2
	Presentation preparation	357	63.3
	Market or industry research	337	59.8
	Problem-solving support	318	56.4
	Strategic idea generation	292	51.8
	Proposal writing	274	48.6
	Client communication	248	44.0
	Benchmarking	211	37.4

**Table 5:** Construct-Level Descriptive Statistics and Intercorrelations.

Construct	Mean	SD	1	2	3	4	5	6	7	8	9	10	11
1. SI	3.58	0.66	1										
2. EI	3.44	0.64	0.32 ***	1									
3. AIM	3.64	0.62	0.21 ***	0.23 ***	1								
4. PS	3.27	0.67	0.36 ***	0.27 ***	0.19 ***	1							
5. HAIT	3.35	0.67	0.11 **	0.12 **	0.35 ***	0.13 **	1						
6. AIPC	3.08	0.67	0.21 ***	0.21 ***	0.13 **	0.31 ***	0.17 ***	1					
7. OAIT	3.18	0.65	0.20 ***	0.22 ***	0.20 ***	0.26 ***	0.25 ***	0.38 ***	1				
8. AIDA	2.86	0.64	−0.26 ***	−0.39 ***	−0.11 **	−0.32 ***	−0.07	−0.33 ***	−0.14 ***	1			
9. CPF	2.76	0.64	−0.29 ***	−0.26 ***	−0.09 *	−0.37 ***	−0.03	−0.20 ***	−0.11 **	0.37 ***	1		
10. RAID	3.30	0.67	0.33 ***	0.22 ***	0.38 ***	0.26 ***	0.33 ***	0.39 ***	0.36 ***	−0.30 ***	−0.14 **	1	
11. HGAI	2.56	0.65	−0.34 ***	−0.27 ***	−0.14 ***	−0.33 ***	−0.10 *	−0.36 ***	−0.28 ***	0.47 ***	0.44 ***	−0.39 ***	1

Note. SI = Social intelligence; EI = Emotional intelligence; AIM = AI metacognition; PS = Psychological safety; HAIT = Human–AI trust; AIPC = AI policy clarity; OAIT = Official AI tool adequacy; AIDA = AI disclosure anxiety; CPF = Competence penalty fear; RAID = Responsible AI disclosure; HGAI = Hidden generative AI use. * *p* < .05, ** *p* < .01, *** *p* < .001.

**Table 6:** CFA model fit and common-method diagnostics.

Diagnostic/Model	χ^2^/Result	CFI	TLI	RMSEA	SRMR	Interpretation
11-factor CFA	1583.17 (df = 1484)	0.992	0.991	0.011	0.032	Excellent fit
Single-factor CFA	8470.30 (df = 1539)	0.416	0.395	0.089	0.104	Poor fit
RAID-HGAI two-factor CFA	40.81 (df = 43)	1.000	1.001	0.000	0.025	Excellent fit
RAID-HGAI one-factor CFA	1015.04 (df = 44)	0.586	0.482	0.198	0.250	Poor fit
Harman first factor	19.4%					No dominant single factor
Full-collinearity VIF	1.232–1.670					All < 3.3

**Table 7:** HTMT values.

Construct	SI	EI	AIM	PS	HAIT	AIPC	OAIT	AIDA	CPF	RAID	HGAI
SI	1										
EI	0.390	1									
AIM	0.253	0.280	1								
PS	0.428	0.322	0.224	1							
HAIT	0.135	0.152	0.418	0.153	1						
AIPC	0.252	0.257	0.156	0.366	0.200	1					
OAIT	0.249	0.271	0.246	0.320	0.308	0.459	1				
AIDA	0.314	0.484	0.136	0.393	0.088	0.402	0.172	1			
CPF	0.355	0.326	0.121	0.451	0.080	0.250	0.161	0.462	1		
RAID	0.399	0.271	0.453	0.315	0.391	0.464	0.431	0.368	0.167	1	
HGAI	0.406	0.320	0.170	0.395	0.116	0.425	0.336	0.567	0.528	0.464	1

**Table 8:** Structural-model robustness across PLS-SEM and CB-SEM.

Hypothesis	Path	PLS β	CB-SEM β	PLS Bootstrap 95% CI	Conclusion
H1	SI → HGAI	−0.105	−0.090	[−0.173, −0.036]	Robust
H2	SI → RAID	0.189	0.212	[0.113, 0.259]	Robust
H3	EI → AIDA	−0.340	−0.415	[−0.415, −0.261]	Robust
H4	AIDA → HGAI	0.272	0.324	[0.199, 0.351]	Robust
H5	AIM → RAID	0.229	0.254	[0.152, 0.305]	Robust
H6	AIM → HAIT	0.352	0.423	[0.275, 0.421]	Robust
H7	HAIT → RAID	0.149	0.161	[0.077, 0.219]	Robust
H8	PS → CPF	−0.370	−0.475	[−0.441, −0.290]	Robust
H9	CPF → HGAI	0.265	0.324	[0.198, 0.342]	Robust
H10	AIPC → AIDA	−0.260	−0.311	[−0.337, −0.179]	Robust
H11	AIPC → RAID	0.241	0.284	[0.167, 0.315]	Robust
H12	OAIT → HGAI	−0.120	−0.120	[−0.191, −0.048]	Robust
H13	OAIT → RAID	0.141	0.142	[0.063, 0.220]	Robust
H14	RAID → HGAI	−0.197	−0.225	[−0.273, −0.116]	Robust

**Table 9:** Explanatory power and predictive relevance.

Endogenous Construct	PLS R^2^	Adjusted R^2^	PLS Q^2^	CB-SEM R^2^
Human–AI trust	0.124	0.122	0.118	0.179
AI disclosure anxiety	0.221	0.218	0.216	0.336
Competence penalty fear	0.137	0.135	0.132	0.226
Responsible AI disclosure	0.343	0.337	0.324	0.440
Hidden generative AI use	0.392	0.386	0.380	0.471

**Table 10:** Effect-size results.

Predictor	Outcome	f^2^	Interpretation
AI metacognition	Human–AI trust	0.141	Small
Emotional intelligence	AI disclosure anxiety	0.141	Small
AI policy clarity	AI disclosure anxiety	0.083	Small
Psychological safety	Competence penalty fear	0.159	Medium
Social intelligence	Responsible AI disclosure	0.049	Small
AI metacognition	Responsible AI disclosure	0.067	Small
Human–AI trust	Responsible AI disclosure	0.029	Small
AI policy clarity	Responsible AI disclosure	0.074	Small
Official AI tool adequacy	Responsible AI disclosure	0.024	Small
Social intelligence	Hidden generative AI use	0.015	Very small
AI disclosure anxiety	Hidden generative AI use	0.096	Small
Competence penalty fear	Hidden generative AI use	0.095	Small
Official AI tool adequacy	Hidden generative AI use	0.020	Small
Responsible AI disclosure	Hidden generative AI use	0.049	Small

**Table 11:** Cross-validated continuous HGAI prediction performance.

Model	MAE Mean	MAE SD	RMSE Mean	RMSE SD	R^2^ Mean	R^2^ SD
Mean baseline	0.528	0.025	0.648	0.023	−0.019	0.020
Ridge regression	0.422	0.032	0.521	0.037	0.341	0.077
Random forest	0.436	0.027	0.538	0.033	0.297	0.064
XGBoost	0.431	0.032	0.536	0.038	0.301	0.079

**Table 12:** Binary HGAI threshold sensitivity and calibration.

Threshold	Model	Positive Cases	AUC	PR-AUC	Precision	Recall	Brier	Cal. Intercept	Cal. Slope
2.50	Logistic	330 (58.5%)	0.782	0.815	0.738	0.794	0.186	−0.004	0.966
2.50	XGBoost	330 (58.5%)	0.760	0.802	0.734	0.794	0.194	−0.012	0.931
3.00	Logistic	174 (30.9%)	0.769	0.582	0.647	0.431	0.173	−0.068	0.893
3.00	XGBoost	174 (30.9%)	0.753	0.567	0.624	0.420	0.178	−0.053	0.922
3.50	Logistic	54 (9.6%)	0.819	0.386	0.500	0.130	0.076	−0.090	0.912
3.50	XGBoost	54 (9.6%)	0.821	0.377	0.500	0.074	0.074	−0.043	0.985

**Table 13:** Out-of-fold SHAP predictor importance for continuous HGAI.

Rank	Predictor	Mean |SHAP|	Relative Importance (%)	Direction
1	AI disclosure anxiety (AIDA)	0.164	32.9	Higher → higher HGAI
2	Competence penalty fear (CPF)	0.120	24.2	Higher → higher HGAI
3	AI policy clarity (AIPC)	0.063	12.7	Higher → lower HGAI
4	Social intelligence (SI)	0.053	10.7	Higher → lower HGAI
5	Official AI tool adequacy (OAIT)	0.051	10.3	Higher → lower HGAI
6	Psychological safety (PS)	0.031	6.2	Higher → lower HGAI
7	AI metacognition (AIM)	0.010	2.0	Weak/generally lower
8	Human–AI trust (HAIT)	0.003	0.7	Weak/generally lower
9	Emotional intelligence (EI)	0.002	0.3	Weak/generally lower

**Table 14:** Evidence-informed MCDM criterion weights (λ = 0.50 baseline).

Rank	Code	Criterion	SEM Weight	SHAP Weight	λ = 0.50 Weight
1	AIDA	AI disclosure anxiety	0.216	0.329	0.273
2	CPF	Competence penalty fear	0.216	0.242	0.229
3	AIPC	AI policy clarity	0.110	0.127	0.119
4	OAIT	Official AI tool adequacy	0.101	0.103	0.102
5	SI	Social intelligence	0.092	0.107	0.100
6	PS	Psychological safety	0.103	0.062	0.083
7	EI	Emotional intelligence	0.090	0.003	0.047
8	AIM	AI metacognition	0.048	0.020	0.034
9	HAIT	Human–AI trust	0.024	0.007	0.016

**Table 15:** Structured intervention performance matrix.

Alternative	AIDA	CPF	AIPC	SI	OAIT	PS	AIM	HAIT	EI
A1 Clear AI disclosure policy	0.75	0.50	1.00	0.25	0.25	0.50	0.25	0.25	0.25
A2 Non-punitive disclosure system	1.00	1.00	0.75	0.50	0.25	1.00	0.25	0.50	0.50
A3 Psychological safety training	0.75	1.00	0.25	0.50	0.00	1.00	0.25	0.50	0.75
A4 Managerial AI literacy training	0.50	0.75	0.75	0.50	0.50	0.75	0.75	0.75	0.50
A5 Official secure AI tools	0.25	0.25	0.50	0.00	1.00	0.25	0.50	0.75	0.00
A6 Responsible AI-use guidelines	0.75	0.50	1.00	0.50	0.50	0.50	0.75	0.50	0.25
A7 AI metacognition/verification training	0.25	0.25	0.25	0.25	0.25	0.25	1.00	0.75	0.50
A8 Client transparency protocol	0.75	0.50	0.75	0.75	0.25	0.50	0.50	0.50	0.50

Note. AIDA = AI disclosure anxiety; CPF = Competence penalty fear; AIPC = AI policy clarity; SI = social intelligence; OAIT = official AI tool adequacy; PS = psychological safety; AIM = AI metacognition; HAIT = human–AI trust; EI = emotional intelligence.

**Table 16:** Baseline Choquet intervention ranking.

Rank	Alternative	Managerial Intervention	Choquet Priority Score
1	A2	Non-punitive AI-use disclosure system	0.760
2	A3	Psychological safety training	0.615
3	A4	Managerial AI literacy training	0.613
4	A6	Responsible AI-use guidelines	0.611
5	A8	Client transparency protocol	0.585
6	A1	Clear AI disclosure policy	0.530
7	A5	Official secure AI tools	0.321
8	A7	AI metacognition and verification training	0.292

**Table 17:** Weighting sensitivity across λ values.

λ (SEM Emphasis)	Ranking	Spearman ρ vs. λ = 0.50
0.00	A2 > A6 > A3 > A4 > A8 > A1 > A5 > A7	0.929
0.25	A2 > A6 > A3 > A4 > A8 > A1 > A5 > A7	0.929
0.50	A2 > A3 > A4 > A6 > A8 > A1 > A5 > A7	1.000
0.75	A2 > A4 > A3 > A6 > A8 > A1 > A5 > A7	0.976
1.00	A2 > A4 > A3 > A6 > A8 > A1 > A5 > A7	0.976

Note. λ represents the relative emphasis placed on normalized SEM evidence; (1 − λ) represents the emphasis placed on normalized out-of-fold SHAP evidence.

**Table 18:** Monte Carlo rank-acceptability results.

Alternative	P (Rank 1) Moderate (%)	P (Top 3) Moderate (%)	P (Rank 1) ± 0.25 Score (%)	P (Top 3) ± 0.25 Score (%)
A2	100.0	100.0	85.1	98.9
A3	0.0	63.9	3.2	—
A4	0.0	62.9	5.4	—
A6	0.0	54.9	3.8	—
A8	0.0	18.2	2.3	—
A1	0.0	<1	0.0	—
A5	0.0	0.0	0.0	—
A7	0.0	0.0	0.0	—

**Table 19:** Additive versus Choquet ranking comparison.

Model	Ranking	Interpretation
Additive weighted model	A2 > A3 > A6 > A4 > A8 > A1 > A5 > A7	Interactions change middle ranks only
Choquet model	A2 > A3 > A4 > A6 > A8 > A1 > A5 > A7	A2 remains first; A4 and A6 exchange positions

## Data Availability

The original contributions presented in this study are included in the article/Supplementary Material. Further inquiries can be directed to the corresponding author.

## Supplementary Materials

The following supporting information can be downloaded at: https://www.mdpi.com/article/10.3390/jintelligence14090196/s1, Table S1: Survey Items and Construct Sources; Table S2: Construct Distribution and Missingness Diagnostics; Table S3: Item-Level Measurement Results and CFA Loadings; Table S4: Bootstrap HTMT Confidence Intervals and Fornell–Larcker Matrix; Table S5: Additional Structural Robustness Analyses; Table S6: Additional Predictive Sensitivity Analyses.
